# Optical Channel Selection Avoiding DIPP in DSB-RFoF Fronthaul Interface

**DOI:** 10.3390/e23111554

**Published:** 2021-11-22

**Authors:** Zbigniew Zakrzewski

**Affiliations:** Institute of Telecommunications and Computer Science, Bydgoszcz University of Science and Technology, Al. Prof. Sylwestra Kaliskiego 7, 85-796 Bydgoszcz, Poland; zbizak@pbs.edu.pl

**Keywords:** fronthaul, Xhaul, DSB-RFoF, A-RoF, B5G, 6G, DIPP, optical channel selection

## Abstract

The paper presents a method of selecting an optical channel for transporting the double-sideband radio-frequency-over-fiber (DSB-RFoF) radio signal over the optical fronthaul path, avoiding the dispersion-induced power penalty (DIPP) phenomenon. The presented method complements the possibilities of a short-range optical network working in the flexible dense wavelength division multiplexing (DWDM) format, where chromatic dispersion compensation is not applied. As part of the study, calculations were made that indicate the limitations of the proposed method and allow for the development of an algorithm for effective optical channel selection in the presence of the DIPP phenomenon experienced in the optical link working in the intensity modulation–direct detection (IM-DD) technique. Calculations were made for three types of single-mode optical fibers and for selected microwave radio carriers that are used in current systems or will be used in next-generation wireless communication systems. In order to verify the calculations and theoretical considerations, a computer simulation was performed for two types of optical fibers and for two selected radio carriers. In the modulated radio signal, the cyclic-prefix orthogonal frequency division multiplexing (CP-OFDM) format and the 5G numerology were used.

## 1. Introduction

The 4G and 5G cellular systems have gained independence in using the domain of packet switching. Bypassing telephone centrals when setting up connections has made it possible to provide broadband services based on wireless access. This kind of approach has resulted in an exponential growth in demand for packet mobile services, which were previously only provided over wired networks. It quickly turned out that the approach to designing mobile systems needed to be changed, as the architecture known from the first generations was no longer effective. As a result of the evolutionary changes, the boundary between the core part of the system and the section with radio resource management and multilevel processing of radio signals from baseband and beyond has been clearly marked.

The original architecture of the network in the radio domain of the distributed radio access network (D-RAN) type, also known as an all-in-one macro base station, provides simplicity, as it consists in constructing base stations as single-device systems for processing and broadcasting the radio signals. The use of such a solution is appropriate when the use of the base station is uniform in time and the peak-load is well below the hardware capabilities of the equipment. As mentioned earlier, the rapidly growing demand for broadband Internet access has changed the paradigm in the approach to designing new wireless communication systems, especially those that can be used to provide more dynamic and sophisticated network services. In order to eliminate the disadvantages of the D-RAN solution, the centralized/cloud radio access network (C-RAN) architecture was introduced. In this solution, the user equipment (UE) is set at the center, which means that the provision of coordinated multi-point (CoMP) services becomes much simpler as spatial access aggregation can take place at the level of one base station. In addition, coverage of the area with a radio signal is much more effective, because the central unit, often located in a computing cloud, has greater coordination possibilities and easier access to the often different needs related to the software signal processing.

The C-RAN concept was used in mobile networks from the second generation (2G), and the architecture related to it was disseminated only at the beginning of the 21st century in order to increase the efficiency of next-generation cellular networks. In all cases, the implementation of this concept required the use of specific media that made it possible to transfer pre-prepared radio signals from a central unit (CU) to a radio unit (RU). With the current advances in transmission technologies, this can be done with a wireless microwave medium or a fiber-optic medium. The microwave radio-link was sufficient for the distribution of signals from 2G and 3G interfaces. The appearance on the global market of 4G systems solely with the domain of packet switching has revolutionized the approach to the methods of modernizing the C-RAN architecture. Optical fiber and networks using its transmission potential appeared in the foreground. In the case of 5G mobile systems, in both the core and radio domains, it is difficult to imagine the functioning of such demanding networks without solutions based on optical fibers. The concept of the Xhaul network [1,2] proposed in the 5GPPP project shows exceptionally well the degree of integration of the fiber-optic and radio domains, which is widely used in 5G solutions [3].

In the paper, will pay particular attention to the use of fiber-optic networks in the radio domain of next generations of mobile systems. Next-generation radio access networks (NG-RANs) are very much based on optical networks, which ensures the construction of efficient and broadband interfaces for backhaul (BH), midhaul (MH) and fronthaul (FH) links. Backhaul networks are a very important broadband link between the core network (5GC) and the NG-RAN domains. They are purely digital networks for the delivery of content for final distribution over the wireless mobile radio links. Midhaul and fronthaul networks are used somewhat differently, as they act as networks distributing digital control signals and radio signals with varying degrees of processing. At this point, the radio-over-fiber (RoF) technique [4] is used, which consists in transmitting radio signals from the baseband (BBoF), intermediate-frequency (IFoF) and radio-frequency (RFoF) over an optical path or optical link, such as an optical fiber [5]. Such a method of increasing the transmission range of a radio signal was first defined in [6], where a unit for introducing a radio signal onto an optical carrier and a unit for inverting the process are defined. Radio signals can be transported over a fiber-optic medium in a digitized version, in the digitized-radio-over-fiber (D-RoF) technique, or in an analog version, in the analog-radio-over-fiber (A-RoF) technique [7].

The D-RoF technique is widely used in radio access systems and in microwave transmission systems. In this respect, the most recognizable is the common public radio interface (CPRI) [8], which has monopolized the market in this field and allows the delivery of digitized radio baseband signals. This interface produces very high bit rates, which is noticeable in the case of antenna installations based on multi-element matrices [9,10]. In order to eliminate the monotype of the D-RoF interface, new solutions were introduced to increase the dynamics of this link. The most recognizable dynamic D-RoF interfaces include eCPRI [11] and the next generation fronthaul interface (NGFI) [12]. An initial outline of these solutions was presented by 3GPP [13] and 5GPPP [3] for applications in the 5G mobile systems. Both solutions were prepared so that radio signal transport services could be implemented in the radio-over-ethernet (RoE) mode. This is a good and optimal complement to the shortcomings of CPRI but requires the use of synchronous packet networks [14,15,16,17]. The popularization of the implementation of the NGFI family interfaces in 5G systems initiated the trend of looking for solutions that reduce the costs of transporting the digitized radio signals prepared in the edge cloud to an active antenna unit or remote radio head (AAU/RRH) [18]. This resulted in the emergence of a new concept of an open radio access network (O-RAN) [19]. The main players in this field dealing with standardization include O-RAN Alliance, 3GPP and IEEE. The openness of mobile networks in the radio domain ensures the cooperation of 5G systems with local and regional networks belonging to various local operators but meeting specific requirements at the level of real-time packet transport. Fulfilling these requirements is more difficult the higher the split/option of the supported network interface is [11,12].

The A-RoF technique is still used in the field of research, as it is based on the transport of signals over optical fibers in the original version. Such a solution is particularly useful when there is a need to transmit a highly processed radio signal at the intermediate carrier (IFoF) or radio broadcast carrier (RFoF) level. The technique of analog transport of radio signals can be implemented in the fiber-to-the-antenna (FTTA) architecture or with the use of an all-optical transport network. Proposed solutions enabling the simultaneous operation of D-RoF and A-RoF interfaces are presented in [20,21]. Optical systems using coarse/dense WDM (C/DWDM) techniques with all-optical nodes and elastic optical networks (EONs) provide a very good network structure suitable for transporting A-RoF signals. On this basis, it can be concluded that all-optical networks fit well with the concept of O-RAN because their appearance in the local and regional domains ensures the transport of optical signals modulated in any modern and classic formats, as long as they are compatible with the grid of optical channels of a given link.

**Figure 1:**
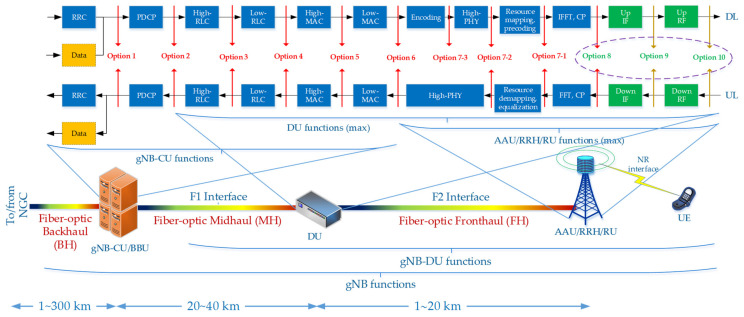
Functional splits proposed by 3GPP [13] in NG-RAN with an example of IF/RF extensions proposed by the author for A-RoF functions introduced into the distributed unit (DU) and the radio unit (RU) (green blocks and options) [21]. Optical BH/MH/FH and their maximal links could be realized in mobile 5G systems [40].

The transmission of optical signals in the A-RoF technique is very spectral-effective and allows signals to be built up on the edge of the network or in the edge cloud to the final form (software defined radio (SDR) technique). The mast with antennas operating in AAU mode is, in this case, a simple device for amplifying and radiating the RF signal. This solution also fits very well with digital (DBF), analog (ABF) and hybrid (HBF) beamforming on the wireless side of the network. However, it should be taken into account that the signal coming from the A-RoF interface has the structure of an analog signal and is strongly influenced by phenomena occurring in the optical path. In the work [21], the author proposed the introduction of two new Splits/Options, in relation to the 3GPP model [13] to support transport in the IFoF (Option 9) and RFoF (Option 10) formats (Figure 1). In the future B5G/6G mobile systems, it is planned to use high millimeter frequencies up to 100 GHz. High radio frequency provides mass and broadband radio access, but over a not very large area. Transporting the radio signals in the RFoF format at such a high radio frequency requires special measures, as the optical path is dispersive. We mean sections of links or optical paths, which should not exceed 20 km, but the influence of phenomena, especially related to the relative delay, is noticeable. Massive access and local or regional use of the RFoF interface necessitates the use of simple and the same modulation and demodulation techniques, so the solution based on IM-DD modulation seems to be optimal. During the IM modulation, a signal in DSB format appears. The sidebands carry information, but at the point of the direct detection (DD) receiver, they are subject to interference, significantly lowering the receiver sensitivity. This phenomenon is strictly dependent on the RF radio frequency, the length of the optical fiber path and the chromatic dispersion coefficient occurring in the optical channel.

For the first time, the effect of the dispersion-induced power penalty (DIPP) phenomenon on the reception of an optical signal in the DSB format was presented in [22]. This was confirmed in many subsequent experiments [23,24,25,26,27]. In the meantime, new formats for modulating radio signals have emerged, and the demand for RoF solutions for use in mobile wireless communication systems has increased. Methods and techniques have been presented in many publications to mitigate or compensate for the effect of DIPP on the efficient reception of modulated optical signals carrying microwave radio signals. The publication of [28] proposed a technique to mitigate the influence of DIPP by appropriate biasing of the electrooptical Mach–Zehnder modulator (MZM) either at the maximum or at the minimum transmission bias points of the operation of an external MZM. The authors of [29] proposed the use of an FBG taper, whose task is narrowband compensation of chromatic dispersion. This is a good solution, but in most optical paths, it is implemented in a static manner with dispersion-compensating fiber (DCF). In the case of fiber-optic access networks, chromatic dispersion compensation is usually not used, which requires it to be taken into account when designing the link. An alternative to static compensators can be dynamic tunable dispersion–compensation module (TDCM) compensation [30,31]. Single-sideband (SSB) modulation was proposed in [32,33] as an effective way to avoid the sideband interference effect at the photodetector point. This method, however, increases the complexity of the modulator system and lowers the sensitivity of the direct photodetection. A method of mitigating the influence of the DIPP was also proposed by appropriately selecting the modulation index and introducing phase imbalance in the signal modulated in the dual-sideband optical carrier suppression (DSB-OCS) format [34]. Another way to eliminate the phenomenon of deep sideband interference in the photodetector can be the introduction of interleaving light intensity modulation (IM-DD) with phase modulation (PM-DD) [35,36].

In this paper, we propose a solution based on a deep analysis of the DIPP interference characteristics and the introduction of a tunable optical carrier source, according to the flexible DWDM system grid [37]. Such an approach requires the use of a suitable transceiver insert on the optical transmitter side with the ability to tune the laser. If the signal is introduced into the optical path of the DWDM network, the laser tuning must follow the system’s optical channel grid, which fits well with the Xhaul architecture supported by the Open RAN Alliance [38]. At the moment, this solution cannot be classified as cheap, but it should be assumed that the increased demand for the development of laser techniques and the wide implementation of flexible DWDM systems in the Xhaul domain will mean that transceivers/transponders equipped with lasers working in accordance with the DWDM grid will in time cease to be a luxury product. The results of the first studies in this area were presented at the international scientific conference “Optical Fibers and Their Applications”, which took place in Poland in 2020 [39].

The paper consists of six sections. The first section presents a broad introduction showing the advisability of researching the optical signals modulated in the A-RoF format, with the rapidly developing D-RoF format. Section 2 describes the system, the methods used, and the network components. In this section, Section 2.1 gives a detailed description of the fiber-optic fronthaul path parameters, and Section 2.2 presents the theoretical basis for the DSB–RFoF signal. Section 3 is devoted to the description of the DIPP phenomenon and the analysis of the interference characteristics formed at the photo-receiver side with direct detection. This section is divided into four subsections, where Section 3.1 deals with the calculations related to the DIPP phenomenon occurring in the Option 10 interface, Section 3.2 deals with the calculations related to the selection of the optical channel for the vRF service, Section 3.3 contains the calculations of the relative delay within the CP-OFDM symbol, and Section 3.4 is devoted to the proposed algorithm for selecting an optical channel. Section 4 presents the results of the verification simulations carried out using the VPIphotonics platform, which show the possibilities and limitations of the proposed “Option 10” [21] split working in the DSB–RFoF format. The last two sections present a broad discussion of the obtained results of calculations and simulations, as well as conclusions and predictions for further research.

## 2. System, Methods, and Network Components

Modern mobile 5G systems are based, in the radio domain, on the C-RAN architecture, strongly promoted by the Open RAN Alliance. It is highly likely that this type of architecture will be the basic determinant of the development of B5G/6G systems and networks. The study below is based on the 3GPP model [13], in which eight basic Splits/Options and three intermediate Splits/Options were defined, which enable the split of functions between network devices due to the processing of information and signals at the level of the NG-RAN domain. The assignment of appropriate functions depends on the current network load and the processing load of individual nodal components. It should be noted here that, in the case of real-time services, e.g., from the ultra-reliable low-latency communication (URLLC) family, it should be ensured that as many stages of information and signal preparation as possible are carried out very close to the place where the radio signal is emitted. On the other hand, the simplicity of the network components located right next to the antenna array provides a flexible approach to network reconfiguration, as most signal processing can be performed in an edge cloud.

Figure 1 shows the functional split of NG-RAN work according to the structure proposed by 3GPP [13]. In order to introduce interfaces from the A-RoF family, two new Splits (Option 9 and Option 10) and the extension of the functions of split number 8 (Option 8) [21] were proposed. When Option 10 is used in the fronthaul link, then the optical signal is in the RFoF format. In Ref. [39], this service is referred to as the virtual radio frequency (vRF), as it provides full virtualization of the site of software building the radio signal to the level of the radio frequency (RF) carrier. Transporting the RF signal over optical fiber is possible only in the fronthaul network link, the length of which cannot exceed 20 km [40]. When we use the Xhaul architecture and the proposed Option 10 split, the RF signal can be transported over various generations of the fiber-optic media. The impact of the accidental transmission parameters of a fiber-optic link, due to the possibility of automatic reconfiguration of the optical path, requires a continuous correction of the use of specific optical resources. In Figure 1, a distributed unit (DU) is responsible for software building a radio signal and inserting it into the optical path, which may be a specialized device or a separate process in the edge computing cloud. On the wireless part, the radio signal is built by the user equipment (UE) as a mobile device. The RU device is responsible for the modulation and demodulation of the optical signal, as well as the amplification of the radio signal and its transmission and reception from the wireless path, by using the antenna array.

### 2.1. Fiber-Optic Fronthaul Path

The fiber-to-the-antenna (FTTA) architecture is used in the basic solutions for connecting the DU and RU. In this case, the optical path is homogeneous, and no active devices are used along the path. In the extended version of the FTTA link, a passive optical network (PON) can be used, which causes a deterioration of the optical power balance due to the presence of high loss optical splitters, but it enables the use of the already existing resources of the optical network. The Xhaul is a universal extension of the optical fiber network with active and reconfigurable devices. The radio signal traveling over the all-optical path will reach optical fiber sections with different propagation properties. At this point, choosing the right optical carrier is crucial.

Three generations of single-mode telecommunications optical fibers are used for the calculations and simulations. The most common single-mode fiber standard is ITU-T G.652D [41]. It has a wide range of single modality versus wavelength. Its counterpart in the version resistant to macro-bending is the fiber of the ITU-T G.657A standard [42]. This is an SMF fiber that is increasingly replacing the fibers of the G.652D standard. ITU-T G.655 fiber cables are often used in DWDM systems [43]. These fibers have a narrower range of single modality as a function of wavelength, due to adjustments related to DWDM standardization. The standard defined in two tables, i.e., G.655D and G.655E, are used for calculations.

**Figure 2:**
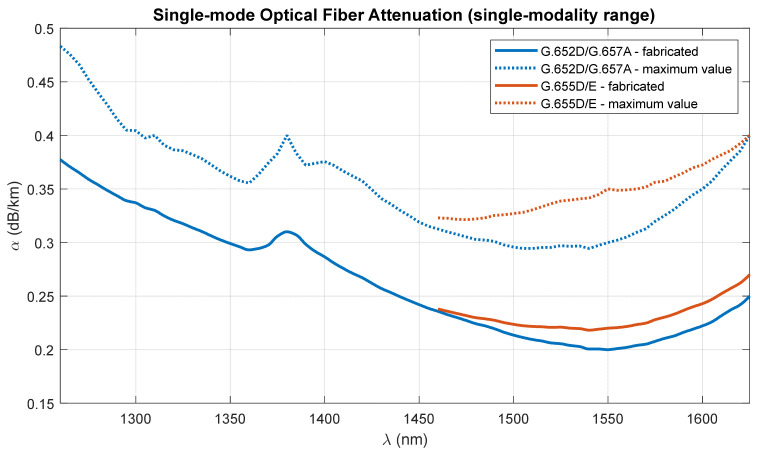
Modeled attenuation characteristics of single-mode optical fibers in range of single-modality limited by the cut-off wavelength [41,42,43].

Table 1 shows the standard and maximum attenuation coefficients of single-mode optical fibers, which were used to determine a more precise absorption characteristic as a function of wavelength [41,42,43,44] (Figure 2). 

**Figure 3:**
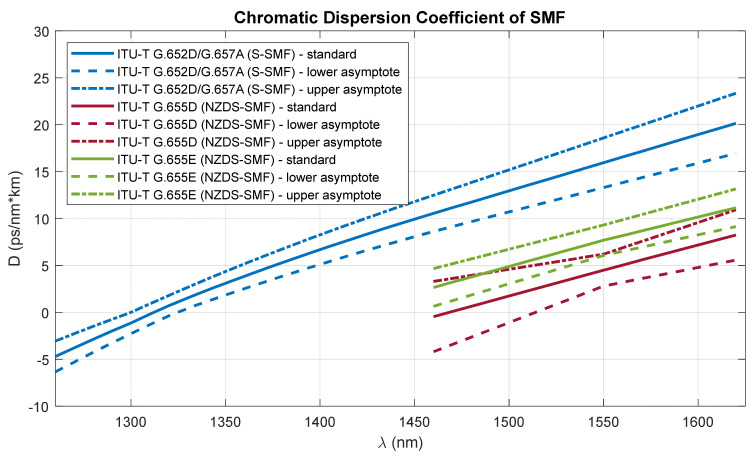
Modeled characteristics of the chromatic dispersion coefficient of the single-mode optical fibers in range of single-modality limited by the cut-off wavelength [41,42,43].

Parameters *D* and *S* were used to model the delay characteristics of the modulated signals. In order to determine the averaged characteristics of the chromatic dispersion coefficient *D* of the G.652D and G.657A standards, the Sellmeier polynomials included in the recommendations [42,43] were used:

(1) $$D_{G.652/G.657}=\left\{\begin{array}{c} \frac{\frac{\lambda S_{0\max}}{4}\left[1-{\left(\frac{\lambda_{0\max}}{\lambda }\right)}^{4}\right]+\frac{\lambda S_{0\min}}{4}\left[1-{\left(\frac{\lambda_{0\min}}{\lambda }\right)}^{4}\right]}{2}\;\mathrm{for}\;1260\;\mathrm{nm}\;\leq \lambda \leq 1300\;\mathrm{nm}\\ \frac{\frac{\lambda S_{0\max}}{4}\left[1-{\left(\frac{\lambda_{0\max}}{\lambda }\right)}^{4}\right]+\frac{\lambda S_{0\max}}{4}\left[1-{\left(\frac{\lambda_{0\min}}{\lambda }\right)}^{4}\right]}{2}\;\mathrm{for}\;1300\;\mathrm{nm}\;<\lambda \leq 1324\;\mathrm{nm}\\ \frac{\frac{\lambda S_{0\min}}{4}\left[1-{\left(\frac{\lambda_{0\max}}{\lambda }\right)}^{4}\right]+\frac{\lambda S_{0\max}}{4}\left[1-{\left(\frac{\lambda_{0\min}}{\lambda }\right)}^{4}\right]}{2}\;\mathrm{for}\;1324\;\mathrm{nm}\;<\lambda \leq 1460\;\mathrm{nm}\\ 0.06\cdot \left(\lambda -1460\right)+10.5485\;\mathrm{for}\;1460\;\mathrm{nm}\;<\lambda \leq 1625\;\mathrm{nm} \end{array}\right.$$

where $\lambda_{0\min}=1300\;\mathrm{nm}$, $\lambda_{0\max}=1324\;\mathrm{nm}$, $S_{0\min}=0.073\;\mathrm{ps}/{\mathrm{nm}}^{2}\cdot \mathrm{km}$, and $S_{0\min}=0.092$ $\mathrm{ps}/{\mathrm{nm}}^{2}\cdot \mathrm{km}$. The characteristics of the chromatic dispersion coefficient of G.655 fibers were modeled using the polynomials contained in the appropriate tables D and E of the recommendation [43] (Figure 3):

(2) $$D_{G.655D}=\left\{\begin{array}{l} \frac{4.955}{90}\left(\lambda -1460\right)-0.455\;\;\mathrm{for}\;1460\;\mathrm{nm}\;\leq \lambda \leq 1550\;\mathrm{nm}\\ \frac{4.015}{75}\left(\lambda -1550\right)+4.500\;\;\mathrm{for}\;1550\;\mathrm{nm}\;<\lambda \leq 1625\;\mathrm{nm} \end{array}\right.$$

(3) $$D_{G.655E}=\left\{\begin{array}{l} \frac{5.035}{90}\left(\lambda -1460\right)+2.650\;\;\mathrm{for}\;1460\;\mathrm{nm}\;\leq \lambda \leq 1550\;\mathrm{nm}\\ \frac{3.710}{75}\left(\lambda -1550\right)+7.685\;\;\mathrm{for}\;1550\;\mathrm{nm}\;<\lambda \leq 1625\;\mathrm{nm} \end{array}\right.$$

Due to the specificity of the operation of flexible DWDM systems, as mentioned before, the average characteristics determined in the wavelength range from 1460 nm to 1625 nm are used for further calculations.

Figure 3 shows the characteristics of the chromatic dispersion coefficients (modeled on the basis of (7)–(9)) for three generations of single-mode fibers, which can be found in the optical fiber network working in the Xhaul architecture. The dashed line defines the upper and lower limits of the chromatic dispersion coefficient that can be found in standard SMFs (S-SMFs) and non-zero dispersion-shifted SMFs (NZDS-SMFs).

Optical fibers of the G.652D standard are the basic medium in optical access networks, so it can be assumed that they are present in cities and their surroundings, but also in rural areas. The G.655D/E optical fibers, which are adapted to DWDM links, are often found between cities and between larger regions. We assume that these fibers appear only in the links between optical nodes that support DWDM streams and very often constitute all-optical nodes as the reconfigurable/optical add-drop multiplexers (OADM/ROADM). The fibers of the recently emerged G.657A generation have parameters very similar to those of the G.652D standard but are more resistant to macro-bending events. As a result, fiber-optic cables with G.657A fibers can be installed in locations with more difficult installation conditions. This type of cabling is an excellent solution when connecting AAU/RRH/RU to CU/DU in FTTA architecture.

**Figure 4:**
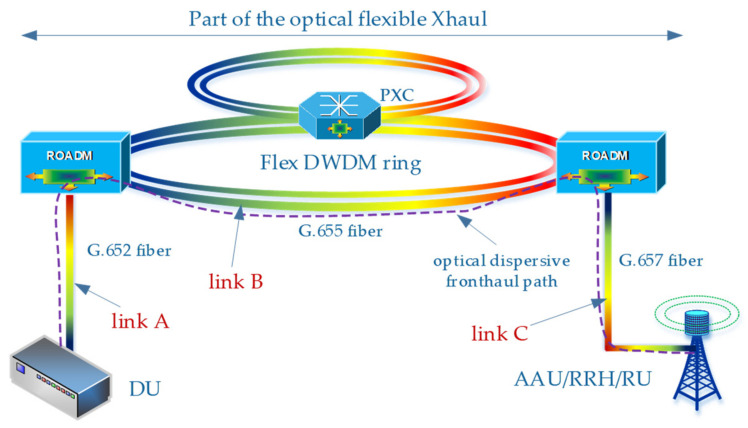
Example of the optical fronthaul path assembled with use of different standards of the single-mode optical fibers.

Figure 4 shows an exemplary slice of the flexible optical Xhaul with fiber access connections. The flexible Xhaul is based on G.655D/E fibers, which guarantees the possibility of providing broadband services with a long range. It should be added that the Xhaul can also provide services in the backhaul domain, which introduces the need to significantly increase the length of optical links (Figure 1). In the access part of the network, on the DU side, standard G.652D fibers are usually used, and on the RRH side, the G.657A standard fibers are preferred. Each of the proposed optical fibers is single mode, so it can carry the traffic of optical signals in accordance with the flexible DWDM channel grid, which is represented in Figure 4 by multi-colored lines connecting network nodes.

The optical path, created as a result of connecting several different optical links, can be characterized by a specific attenuation depending on the carrier wavelength (Figure 2), as well as the averaged chromatic dispersion coefficient (Figure 3). The path attenuation depends not only on the length of the optical fiber used, but also on the number of splices or the physical contact (PC) connectors, the number of ROADM/OADM or passive optical splitters (Table 2). In a network using the flexible DWDM system, we assume that optical splitters are not applicable, but in the case of the access part, such optical systems can be used. In this case, PON networks, which currently constitute the basis for the operation of cable networks and fiber-optic Internet access in FTTH format, come to the rescue. In the part of the optical path, on the DWDM link side, an optical amplifier may be used. Such a system is not taken into account during calculations and simulations due to the significant band limitations introduced in the wavelength domain. If there is an amplifier in the optical path, the choice of the optical channel is significantly limited.

The attenuation of the individual components in the optical path varies with the wavelength of the optical signal carrier. However, the proportions of wavelength-dependent changes are greatest in the case of attenuation of the optical fiber itself. With a short optical path with a length not exceeding 20 km, the number of connections and node devices is not too large. Constant average values provided by the system or device manufacturer may be used in the calculations. 

In order to visualize the changes taking place in the averaged chromatic dispersion coefficient, sample calculations were made for five different variants (Table 3) based on the network diagram in Figure 4.

In all variants, it was assumed that link B is equipped with an optical fiber adapted to work in the DWDM environment, while external access cables are based on standard fibers, which have a higher chromatic dispersion coefficient.

**Figure 5:**
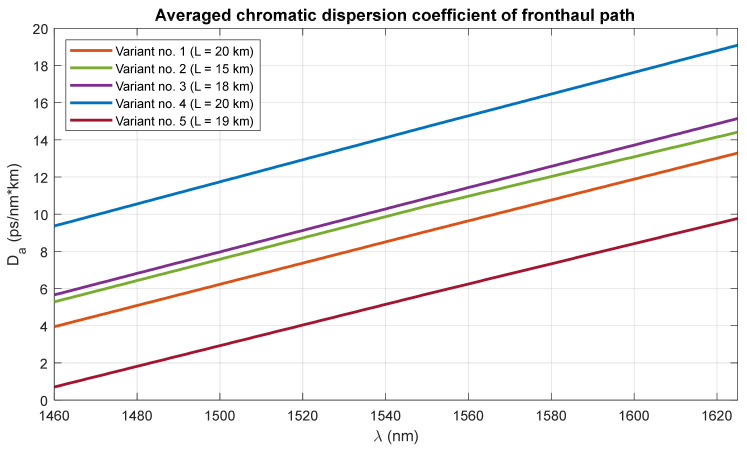
Averaged characteristics of the chromatic dispersion coefficients for the exemplary optical fronthaul paths.

The calculation results presented in Figure 5 show that despite the relatively short optical path, the variability of the averaged chromatic dispersion coefficient, with a large variety of the fibers used, may be significant. The nodal devices are not taken into account in the calculations, as they do not introduce dispersive phenomena large enough to be of significant importance in creating the averaged dispersion characteristic.

### 2.2. Radio-Frequency-over-Fiber in DSB Format

From the three A-RoF interface formats mentioned in the introduction above, the RFoF format is the most susceptible to the dispersion parameters of a single-mode optical fiber. This type of signal is present at the level of the proposed Option 10 interface (Figure 1). In the case of the classic and at the same time the simplest optical IM-DD link, the RFoF signal contains amplitude modulation (AM) products in the continuous version of the carrier. At the beginning of the analysis, we assume that the radio signal consists of a non-modulated single-tone carrier with a frequency $f_{RF}$ that is a signal modulating an optical carrier with wavelength $\lambda_{0}$. In the case of RF signals, we operate with frequency units and in the case of optical signals, with the wavelength units. At a later stage, we move on to the frequency domain also on the optical side.

The signal modulated in the RFoF format, coming from the optical modulator, can be represented by the formula making the optical carrier signal dependent on the RF signal:

(4) $$E_{IM}\left(t\right)=\sqrt{1+m_{IM}\cos\left(2\pi f_{RF}\right)}\exp\left(j2\pi \frac{c}{\lambda_{0}}t\right),$$

where $m_{IM}$ is the intensity modulation index. When $m_{IM}$ is small, the optical field (4) can be written as follows:

(5) $$E_{IM}\left(t\right)=\left(1+m_{IM}\cos\left(2\pi f_{RF}t\right)\right)\exp\left(j2\pi \frac{c}{\lambda_{0}}t\right).$$

If we transform formula (5) trigonometrically, we obtain a relationship showing the frequency components that create the IM signal:

(6) $$E_{IM}\left(t\right)=\exp\left(j2\pi \frac{c}{\lambda_{0}}t\right)+\frac{m_{IM}}{2}\left\{\exp\left[j2\pi \left(\frac{c}{\lambda_{0}}+f_{RF}\right)t\right]+\exp\left[j2\pi \left(\frac{c}{\lambda_{0}}-f_{RF}\right)t\right]\right\}.$$

As we can see in formula (6), the signal takes the form of a DSB, where we can distinguish the optical carrier and two side fringes, which in the case of a modulated RF signal, are sidebands. Now the signal represented by (6) is introduced to the optical path with the dispersive optical fiber, while ignoring the absorption and scattering loss:

(7) $$\begin{array}{c} E_{IM}\left(t,\cdot \right)=\exp\left(j2\pi \frac{c}{\lambda_{0}}t\right)+\frac{m_{IM}}{2}\left\{\exp\left[j2\pi \left(\frac{c}{\lambda_{0}}+f_{RF}\right)t-\Phi \left(\cdot \right)\right]+\right.\\ +\left.\exp\left[j2\pi \left(\frac{c}{\lambda_{0}}-f_{RF}\right)t-\Phi \left(\cdot \right)\right]\right\}. \end{array}$$

In the context of the phenomenon taking place in the photodetector working in the direct detection format, it can be assumed that the optical path is a band-pass filter with a flat amplitude response and a linear group delay factor. On this basis, we can assume that the fiber-optic transfer function can be represented by the following [45]:

(8) $$H\left(f_{RF},\lambda_{0}\right)=\exp\left(-j\Phi \left(f_{RF},\lambda_{0}\right)\right)$$

where

(9) $$\Phi \left(f_{RF},\lambda_{0}\right)=\pi D\left(\lambda_{0}\right)L\frac{\lambda_{0}^{2}}{c}f_{RF}^{2}$$

and $D\left(\lambda_{0}\right)$ is the chromatic dispersion coefficient changing with the optical carrier $\lambda_{0}$ (Figure 3). *L* is the optical path length. After substituting (9) into (7), we obtain the dependence on the electric field strength after the signal passes through the dispersive optical path:

(10) $$\begin{array}{c} E_{IM}\left(t,f_{RF},\lambda_{0}\right)=\exp\left(j2\pi \frac{c}{\lambda_{0}}t\right)+\frac{m_{IM}}{2}\left\{\exp\left[j2\pi \left(\frac{c}{\lambda_{0}}+f_{RF}\right)t-\pi D\left(\lambda_{0}\right)L\frac{\lambda_{0}^{2}}{c}f_{RF}^{2}\right]+\right.\\ +\left.\exp\left[j2\pi \left(\frac{c}{\lambda_{0}}-f_{RF}\right)t-\pi D\left(\lambda_{0}\right)L\frac{\lambda_{0}^{2}}{c}f_{RF}^{2}\right]\right\}. \end{array}$$

The electrical signal behind the photodetector is proportional to the intensity of the incoming light, so the square law detection applies [26,35,36]:

(11) $$i_{IM}\left(t\right)\propto {\left|E_{IM}\left(t,f_{RF},\lambda_{0}\right)\right|}^{2}.$$

When we substitute (10) into (11), we obtain a relationship containing a constant component,$f_{RF}$ dependent components and higher-order components. When we assume that the modulation index $m_{IM}$ is small, the higher-order terms become negligibly small. If we also omit the constant component which does not carry information, the signal takes the following approximate form:

(12) $$i_{IM}\left(t,f_{RF},\lambda_{0}\right)\approx 2m_{IM}\cos\left(\pi D\left(\lambda_{0}\right)L\frac{\lambda_{0}^{2}}{c}f_{RF}^{2}\right)\cos\left(2\pi f_{RF}t\right)$$

Consequently, the normalized power response, downstream of the photodetector, on the excitation of the fiber-optic path by the IM signal, is determined by the following relationship [46]:

(13) $$P_{IM}\left(f_{RF},\lambda_{0}\right)\approx \cos^{2}\left(\pi D\left(\lambda_{0}\right)L\frac{\lambda_{0}^{2}}{c}f_{RF}^{2}\right).$$

Based on formula (13), it can be seen that the power of the received RF radio signal periodically declines to zero. We define this phenomenon as a dispersion induced power penalty (DIPP), and it is the main element of further considerations. After that, we can go to the logarithmic scale to determine the decay ranges of the received RF signal and to combine the DIPP with the absorption loss of the optical path. All components of the optical signal around the $\lambda_{0}$ carrier undergo the same attenuation due to absorption. On this basis, we can determine the degree of fading of the sidebands signal as a result of the DIPP carrier-to-interference ratio (DIPP-CIR) interaction, in relation to the optical carrier as the source of the constant component:

(14) $$\begin{array}{c} {\mathrm{DIPP}-\mathrm{CIR}}_{IM}=10\log\frac{P_{C}}{P_{SBs}}=10\log\cos^{2}\left(\pi D\left(\lambda_{0}\right)L\frac{\lambda_{0}^{2}}{c}f_{RF}^{2}\right)\\ =20\log\cos\left(\pi D\left(\lambda_{0}\right)L\frac{\lambda_{0}^{2}}{c}f_{RF}^{2}\right). \end{array}$$

Given that the optical signal is also subject to absorption loss, the power of the received RF signal downstream of the photodetector is approximately as follows:

(15) $$P_{out}\left(f_{RF},\lambda_{0}\right)\approx P_{in}-{\;\mathrm{DIPP}-\mathrm{CIR}}_{IM}\left(f_{RF},\lambda_{0}\right)-A_{pp}\left(\lambda_{0}\right),$$

where $A_{pp}\left(\lambda_{0}\right)$ is the optical path loss.

## 3. Calculation Results

In the modern 5G-NR radio interface, two frequency ranges are planned to be used, i.e., FR1 [47] to 6 GHz and FR2 [48] to 52.6 GHz. The future B5G/6G wireless systems will also use the higher frequency ranges, i.e., above 60 GHz. For this reason, slightly higher radio frequencies (RFs) are also used in the calculations.

### 3.1. DIPP-CIR in the Option 10 Fronthaul Path

In fiber-optic communications with optical access, transceivers operating at a wavelength of 1310 nm are very often used. In the case of the Xhaul using optical channels supported by the flexible DWDM system, we move to the S, C and L optical bands. In this case, a transponder should be used in the place of connection to the optical fronthaul path, which will convert the wavelength in accordance with the DWDM grid. In the middle of the C band there is an optimal wavelength of 1550 nm for which preliminary calculations were made to show the influence of the DIPP phenomenon.

**Figure 6:**
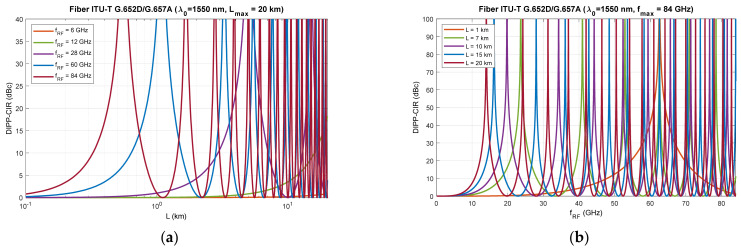
Dispersion induced power penalty—carrier-to-interference ratio obtained after propagation over G.652D or G.657A single-mode fiber and direct detection in photodetector: (**a**) calculated values for the five selected RF carriers as a function of the optical fronthaul path length; (**b**) calculated values for the five selected optical fronthaul path lengths as a function of the RF carrier.

**Figure 7:**
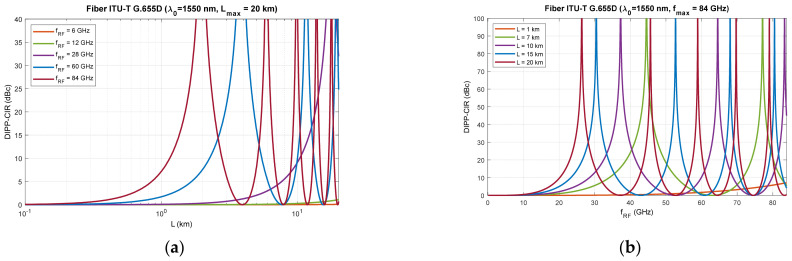
Dispersion induced power penalty—carrier-to-interference ratio obtained after propagation over G.655D single-mode non-zero dispersion-shifted fiber and direct detection in photodetector: (**a**) calculated values for the five selected RF carriers as a function of the optical fronthaul path length; (**b**) calculated values for the five selected optical fronthaul path lengths as a function of the RF carrier.

**Figure 8:**
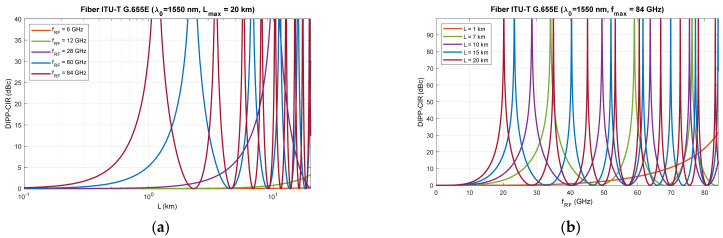
Dispersion-induced power penalty—carrier-to-interference ratio obtained after propagation over G.655E single-mode non-zero dispersion shifted fiber and direct detection in photodetector: (**a**) calculated values for the five selected RF carriers as a function of the optical fronthaul path length; (**b**) calculated values for the five selected optical fronthaul path lengths as a function of the RF carrier.

Figure 6, Figure 7 and Figure 8 show that RF dropouts due to direct detection are periodic both as a function of the optical path length and as a function of the radio frequency, which is the modulating signal. This is a well-known phenomenon but has always been considered in the context of a G.652D-based optical path and a wavelength of 1550 nm as the primary optical carrier.

In Figure 6, we can see the significant effect of G.652D fiber dispersion. In this case, decay occurs quickly, and their periodicity is highly densified. The G.655D/E fiber (Figure 7 and Figure 8) has a much smaller impact on the short periodicity of the interference signal fading. Additionally, in the areas of constructive interference, there are quite flat characteristics, which ensure good transmission of broadband radio signals. This issue is discussed later in the paper.

On the basis of the obtained results, it can also be concluded that the influence of the DSB signal interference phenomenon during direct detection is significant at higher radio frequencies. This mainly applies to the FR2 band [48] intended for use in 5G/B5G systems, which reaches the range of 60 GHz, as well as higher bands, which are adapted in subsequent releases and generations of wireless access systems. The optical path for signal transmission in the RFoF technique should not exceed 20 km (Figure 1), and thus, can be implemented in a passive network, where chromatic dispersion compensation is usually not practiced.

The next stage of calculations is closely related to the dispersion characteristics of single-mode optical fibers of different generations, in relation to the optical channels used in DWDM systems. The single-modality range of G.652D and G.657A fibers is much wider (Figure 3), but due to the limitations of G.655 fibers, the total optical bandwidth (S + C + L) is used for the calculations. This is a practical range of single-mode optical fibers adapted to work with dense wavelength multiplexing systems.

**Figure 9:**
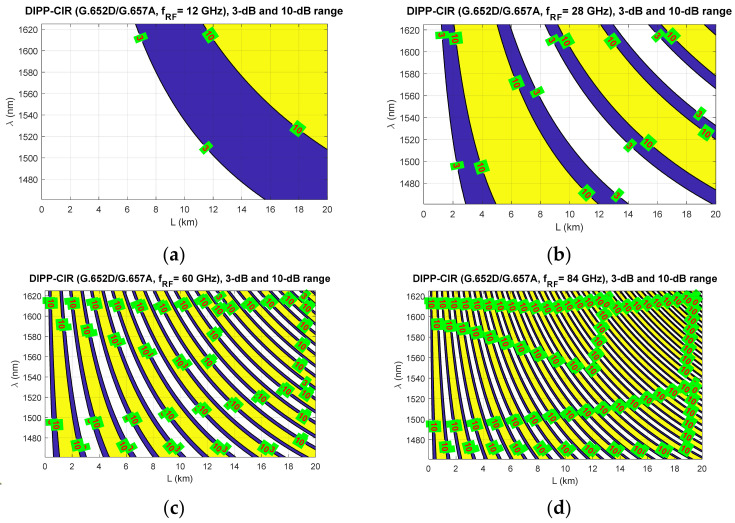
The results of the dispersion-induced power penalty calculations, taking into account the 3 dB and 10 dB thresholds, obtained in the range of the S, C and L optical bands for the fronthaul path based on the G.652D or G.657A optical fiber of variable length: (**a**) $f_{RF}=12\;\mathrm{GHz}$; (**b**) $f_{RF}=28\;\mathrm{GHz}$; (**c**) $f_{RF}=60\;\mathrm{GHz}$; (**d**) $f_{RF}=84\;\mathrm{GHz}$.

**Figure 10:**
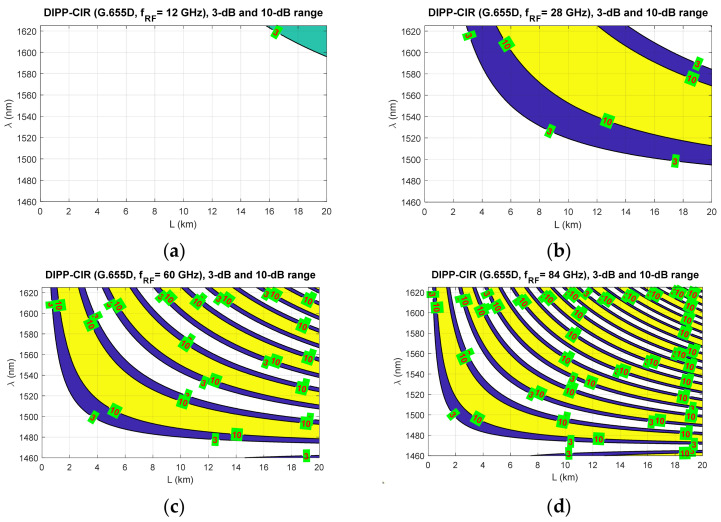
The results of the dispersion-induced power penalty calculations, taking into account the 3 dB and 10 dB thresholds, obtained in the range of the S, C and L optical bands for the fronthaul path based on the G.655D optical fiber of variable length: (**a**) $f_{RF}=12\;\mathrm{GHz}$; (**b**) $f_{RF}=28\;\mathrm{GHz}$; (**c**) $f_{RF}=60\;\mathrm{GHz}$; (**d**) $f_{RF}=84\;\mathrm{GHz}$.

**Figure 11:**
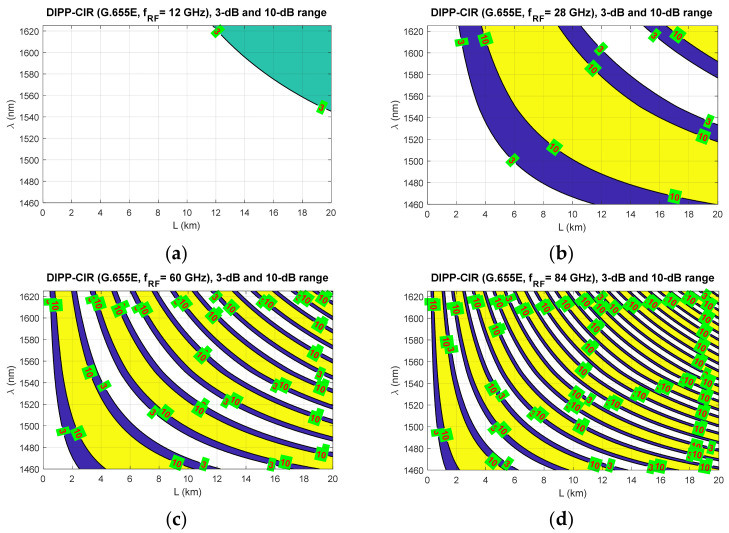
The results of the dispersion-induced power penalty calculations, taking into account the 3 dB and 10 dB thresholds, obtained in the range of the S, C and L optical bands for the fronthaul path based on the G.655E optical fiber of variable length: (**a**) $f_{RF}=12\;\mathrm{GHz}$; (**b**) $f_{RF}=28\;\mathrm{GHz}$; (**c**) $f_{RF}=60\;\mathrm{GHz}$; (**d**) $f_{RF}=84\;\mathrm{GHz}$.

Figure 9, Figure 10 and Figure 11 show the fixed limits of 3 dB and 10 dB that define the areas in the optical wavelength and path length domains. On this basis, it is possible to determine the relationship that classifies the quality of the optical channel (QoOCh) prepared to carry the DSB signal over the optical path in the RFoF format:

(16) $$\mathrm{QoOCh}=\left\{\begin{array}{l} \mathrm{strong}\;\;for\;\;{\mathrm{DIPP}-\mathrm{CIR}}_{IM}<3\mathrm{dB}\\ \mathrm{weak}\;\;for\;\;3\mathrm{dB}<{\mathrm{DIPP}-\mathrm{CIR}}_{IM}<10\mathrm{dB}\\ \mathrm{unusable}\;\;for\;\;{\mathrm{DIPP}-\mathrm{CIR}}_{IM}>10\mathrm{dB} \end{array}\right.$$

The white area visible in Figure 9, Figure 10 and Figure 11 is the best optical carrier range for a particular optical path length. The blue field is a transition region with a fast quenching of the sidebands in the 3–10 dB range. Yellow areas indicate a DIPP-CIR greater than 10 dB, which, combined with the absorption losses, gives ranges of poor transmission quality. It should be noted, however, that in the case of radio carrier modulation with a low order applied, this range may also be partially used.

Figure 9 shows the results of the calculations concerning the DIPP phenomenon occurring in the photodetector after the modulated optical signal has passed over the G.652D or G.657A optical fiber. In this type of fiber, there are yellow areas that cover the entire range of the S + C + L bands. This mainly concerns short sections of several kilometers for radio frequencies from 28 GHz (Figure 9b–d). In the case of the G.655D fiber, the situation is much better (Figure 10) because we can always find the optical range that lies in the white or blue field. The G.655E standard optical fiber is characterized by an indirect characteristic of the chromatic dispersion coefficient (Figure 2), which allows the lack of availability for most radio carrier frequencies to be avoided. The exception here is the case of frequencies above 80 GHz, where for an optical path with a length of about 1.5 km, there may be problems with selecting the appropriate optical channel.

**Figure 12:**
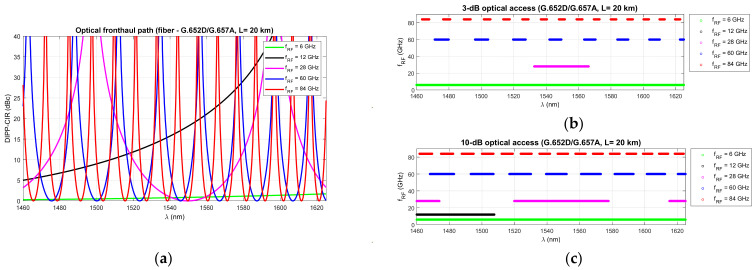
DIPP-CIR as a function of the optical wavelength for selected radio carrier frequencies and the 20 km optical path created on the basis of the G.652D or G.657A optical fiber: (**a**) calculation results without selection; (**b**) 3 dB cut-off optical access ranges; (**c**) 10 dB cut-off optical access ranges.

**Figure 13:**
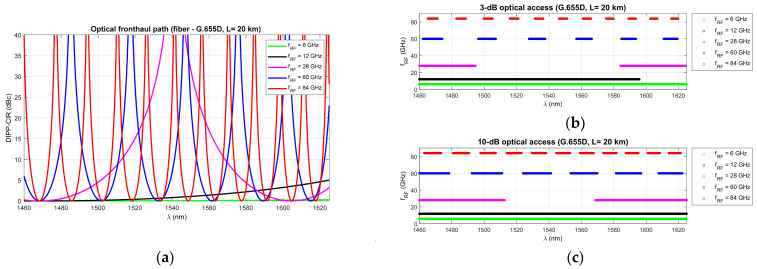
DIPP-CIR as a function of the optical wavelength for selected radio carrier frequencies and the 20 km optical path created on the basis of the G.655D optical fiber: (**a**) calculation results without selection; (**b**) 3 dB cut-off optical access ranges; (**c**) 10 dB cut-off optical access ranges.

**Figure 14:**
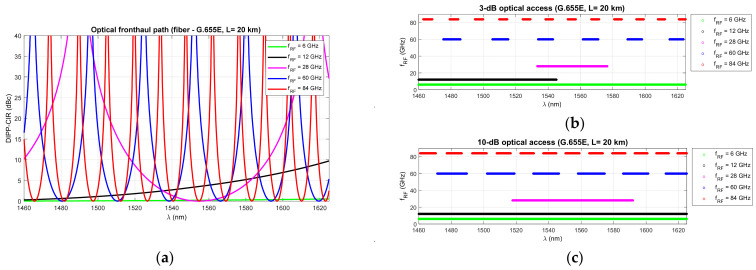
DIPP-CIR as a function of the optical wavelength for selected radio carrier frequencies and the 20 km optical path created on the basis of the G.655E optical fiber: (**a**) calculation results without selection; (**b**) 3 dB cut-off optical access ranges; (**c**) 10 dB cut-off optical access ranges.

Figure 12, Figure 13 and Figure 14 show the results of the DIPP-CIR signals calculated as a function of the optical wavelength. In order to determine the 3 dB and 10 dB optical wavelength range, a cut-off condition was applied. On this basis, we can try to adjust the optical channel for a specific optical path length and for a specific radio carrier frequency. The calculation results presented in Figure 12, Figure 13 and Figure 14 relate to a path with a length of 20 km. Figure 12 shows the results of the DIPP interaction after passing over the G.652D or G.657A fiber. It is significant that for the case *f_RF_* = 12 GHz, no optical band range with a threshold below 3 dB is available (Figure 12b—black color). The only thing left to do here is to select the optical channel in the range between the 3 dB and 10 dB thresholds (Figure 12c). The situation looks much better in the case of the optical path built on the basis of G.655D (Figure 13) and G.655E (Figure 14) fibers. In this case, we can always find optical resources of good quality due to DIPP.

### 3.2. Optical Subband Selection for vRF Service

The bandwidth of the optical channel is strictly dependent on the grid defined in the DWDM system [37]. However, the flexibility of the system allows for uneven reservation of optical resources, which increases the spectral efficiency of the use of the optical path built on the basis of a single-mode optical fiber. The optical carrier can change with a 2 times finer grid (6.25 GHz) in relation to the minimum channel bandwidth, which means that the minimum optical channel bandwidth is 12.5 GHz. In an all-optical fronthaul link, it can be assumed that the nodal devices are insensitive to the modulation format used in the optical channel, but the channel bandwidth and optical carrier must be defined by the optical resource management system (Table 4). ROADM optical access nodes are equipped with filters, which should also be adapted to the supported DWDM grid. Lasers used in optical transmitters are not always characterized by highly stable operation, which causes the phenomenon of optical drift. Hence, the optical modulated signal needs a wider optical channel, both when passing through a filter built into the ROADM node and when transmitting over a DWDM link on the Xhaul side. From here, on the optical side, we also use the unit of frequency, which ensures compliance with the grid given in [37] and makes the specification of the channel width independent of the carrier wavelength.

In the next step, based on relationships (14) and (16), optical subbands (from the S + C + L band range) are determined, in which optical channels can be selected with a step of 6.25 GHz or higher. This depends on the capabilities of the tunable laser, which is built into the optical transmitter, or on the fixed lasers available at the transmitter site. In the case of a low frequency radio carrier where the DIPP interaction is weak, a narrower optical channel should be used (Table 4), and the needs accordingly increase as the frequency of the radio carrier increases. The degree of laser tuning or wavelength selection from the available allows for a much more precise selection of optical resources in the presence of the DIPP. In filling the gaps between the channels, such a dense laser tuning mesh also increases flexibility. Table 5 shows the subbands that provide access to a 20 km fronthaul path based on G.652D or G.657A optical fiber. The 3 dB criterion was not met in the whole wavelength range for the radio signal with *f_RF_* = 12 GHz, which resulted in a lack of access to the service for this link. In other cases, optical resources are available. In a situation where a specific optical path does not have optical resources that can be used to transmit a radio signal with a specific radio frequency, then an attempt should be made to look for an alternative path with a different chromatic dispersion factor *D*. It is advisable that with such a systemic approach, each optical path found should be tested in advance for its accumulated chromatic dispersion. This ensures a quick decision by the algorithm searching for matched optical resources for transporting the RF signal. The tabulated results show that calculations of this kind can be performed for any FH path length and any type of single-mode fiber, also in mixed versions (for example, Figure 5 and Table 3).

Table 6 and Table 7 contain the results of searching for the available optical subbands also for two thresholds, but for optical paths built on the basis of optical fibers of the G.655 family. The optical fiber with a non-zero chromatic dispersion-shifted coefficient is characterized by a much lower dispersion coefficient, which makes the available areas of the optical subbands wider. It can be concluded that in the case of reduced dispersion or partially compensated, e.g., in a link belonging to the DWDM transit domain (for example Figure 4, link B), the number of useful optical channels is much greater.

The calculation results presented in the tables and figures above relate to the transmission of unmodulated radio carrier in the links of the optical fiber network, but do not take into account the bandwidth of the radio frequency channel. The modulation of the radio carrier causes the appearance of frequency components that make up a broadband signal that must be within the range of a specific radio channel. The bandwidth of this channel depends on the modulation format, bit rate and the filtering criterion.

### 3.3. Relative Delay and CP-OFDM Modulation

The CP-OFDM modulation is applied to the 5G-NR downlink, which allows dynamic allocation of radio resources to different user equipment (UE) on the same frequency channel. The smallest unit consists of 12 OFDM subcarriers, and the frequency bandwidth of this resource is dependent on a numerological parameter that defines the frequency spacing between OFDM subcarriers [49,50] (Table 8). As a result of combining all radio resource blocks, a frequency channel is created, which has a width of up to 400 MHz in 5G NR. Of course, the entire channel is not signal covered, as it requires frequency guard intervals [50], which limit the actual frequency bandwidth of the modulated OFDM signal. The maximum channel bandwidths, which are given in Table 8, are taken into account for the calculations. This ensures the consistency of the calculations and better visual analysis resulting from the numerology used in the 5G NR interface.

In order to determine the effect of the channel bandwidth on the OFDM subcarrier transfer unevenness in the RFoF link, we introduce a dependence on the degree of variation in the level of the extreme subcarriers belonging to the RF radio channel. For this purpose, we use formula (14), in which the frequency spacing of the extreme OFDM subcarrier from the middle RF carrier is defined as half the width of the frequency channel:

(17) $$\begin{array}{c} \Delta {\mathrm{DIPP}-\mathrm{CIR}}_{\left(\mathrm{dB}\right)}\left(f_{0},\Delta f_{RF}\right)=\\ \left|{\mathrm{DIPP}-\mathrm{CIR}}_{\left(\mathrm{dBc}\right)}\left(f_{0}-\frac{\Delta f_{RF}}{2}\right)-{\mathrm{DIPP}-\mathrm{CIR}}_{\left(\mathrm{dBc}\right)}\left(f_{0}+\frac{\Delta f_{RF}}{2}\right)\right|. \end{array}$$

The value of ΔDIPP-CIR, determined by relationship (17), is related to the first *Th*1 threshold, which determines the allowed DIPP-CIR value for the carrier frequency *f*_0_ of the optical channel. In order to decide whether the optical channel is suitable for carrying a signal in the DSB-RFoF format, a second threshold, *Th*2, is determined that ultimately limits the use of the designated channel by threshold *Th*1. The *Th*1 and *Th*2 thresholds are used in the algorithm for determining the optical channel subband as a fixed value of the impact of the DIPP phenomenon.

**Figure 15:**
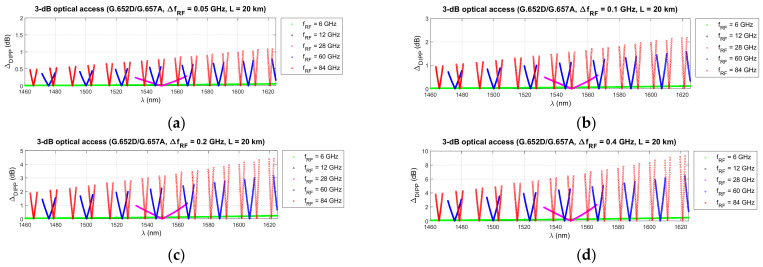
Differential DIPP-CIR as a function of the optical wavelength for selected radio carrier frequencies and the 20 km optical path created on the basis of the G.652D or G.657A optical fiber, and for selected radio channel widths: (**a**) 50 MHz; (**b**) 100 MHz; (**c**) 200 MHz; (**d**) 400 MHz.

**Figure 16:**
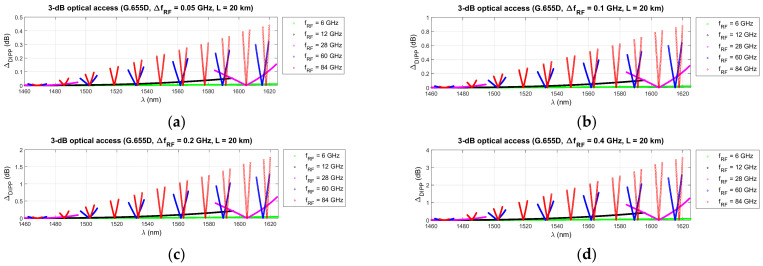
Differential DIPP-CIR as a function of the optical wavelength for selected radio carrier frequencies and the 20 km optical path created on the basis of the G.655D optical fiber, and for selected radio channel widths: (**a**) 50 MHz; (**b**) 100 MHz; (**c**) 200 MHz; (**d**) 400 MHz.

**Figure 17:**
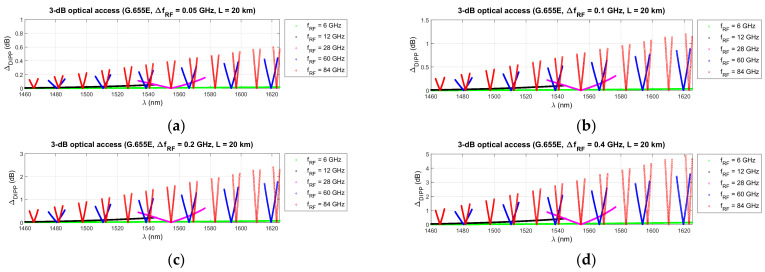
Differential DIPP-CIR as a function of the optical wavelength for selected radio carrier frequencies and the 20 km optical path created on the basis of the G.655E optical fiber, and for selected radio channel widths: (**a**) 50 MHz; (**b**) 100 MHz; (**c**) 200 MHz; (**d**) 400 MHz.

Figure 15, Figure 16 and Figure 17 show the results of the differential DIPP-CIR calculation for the first 3 dB threshold only. The influence of the radio frequency channel bandwidth on the severity of the OFDM subcarrier level differentiation phenomenon is significant. With increasing frequency of the radio carrier, the increase is so large that the selection of the optical channel is possible in very narrow ranges, much narrower than those given in Table 6, Table 7 and Table 8. It should also be noted that not all channels with 100 MHz bandwidth can be used. In the radio band FR1 [50], it is not possible to create wider channels, due to the high fragmentation and dedicated use of subbands. Wider channels realization is only possible in the higher frequency bands in the FR2 range [50]. Thus, it can be seen that not all the combinations, shown in Figure 15, Figure 16 and Figure 17, are currently possible and practical to implement. In the case of the G.655D fiber, the results of the calculations show that practically, the full 3 dB range, according to *Th*1, can be accepted for implementation (Figure 16). In the case of the G.655E fiber, the *Th*2 threshold limitation is necessary for 400 MHz bandwidth channels and a radio carrier with a frequency above 60 GHz (Figure 17d). In this case, the differential DIPP-CIR exceeds 3 dB. When analyzing the graph in Figure 15, we can see that significant limitations appear with a 200 MHz channel, obviously with a carrier frequency above 60 GHz. Here, in the optical path, there is a classic G.652D or G.657A access fiber. Thus, a *Th*1 threshold of 10 dB may have practical applications for bands lying in the FR1 range.

In the next step, we look at the susceptibility of the CP-OFDM symbol to the multipath effect caused by the propagation of two optical sidebands in the DSB format. For this purpose, we determine the dependence on the total width of the optical channel (assuming a very narrow spectral line of the laser in relation to the radio frequency):

(18) $$\Delta f_{0}=2f_{RF}+\Delta f_{RF}$$

where $f_{RF}$ is the central radio frequency of the channel and $\Delta f_{RF}$ is the radio frequency width of the signal containing OFDM subcarriers and side bands of the outer subcarriers. Then we move on to the wavelength domain, while taking into account the optical carrier wavelength that is used during transmission in a fiber of a certain standard. For this purpose, we use the following approximate relationship:

(19) $$\Delta \lambda_{0}=\lambda_{2}-\lambda_{1}=\frac{c\cdot \Delta f_{0}}{\left(f_{2}-\Delta f_{0}\right)\cdot f_{2}}=\frac{c\cdot \Delta f_{0}}{\left(f_{1}+\Delta f_{0}\right)\cdot f_{1}}≅\frac{c\cdot \Delta f_{0}}{f_{0}^{2}}≅\frac{\Delta f_{0}\cdot \lambda_{0}}{c}$$

where $f_{0}=f_{1}+\Delta f_{0}/2=f_{2}-\Delta f_{0}/2$, and $f_{0}=c/\lambda_{0}$. After substituting the dependence (18) into (19), we obtain the following:

(20) $$\Delta \lambda_{0}=\frac{\lambda_{0}\left(2f_{RF}+\Delta f_{RF}\right)}{c}$$

The effective chromatic dispersion coefficient is used to calculate the relative delay as follows:

(21) $$D_{eff}\left(\lambda_{0},\Delta \lambda_{0}\right)=D\left(\lambda_{0}\right)+S\left(\lambda_{0}\right)\cdot \left(\frac{\Delta \lambda_{0}}{2}\right)$$

where *S* is the chromatic dispersion slope. Now, taking into account (21), the relative delay can be determined using the following equation:

(22) $$\tau_{DSB\max}\left(\lambda_{0}\right)=D_{eff}\left(\lambda_{0}\right)\Delta \lambda_{0}L=\left(\left|D\left(\lambda_{0}\right)\right|\cdot \Delta \lambda_{0}+\frac{1}{2}S\cdot \Delta \lambda_{0}^{2}\right)\cdot L.$$

After substituting (20) into (22) we obtain the following:

(23) $$\tau_{DSB\max}\left(\lambda_{0}\right)=\left(\left|D\left(\lambda_{0}\right)\right|\left(2f_{RF}+\Delta f_{RF}\right)+\frac{S\lambda_{0}{\left(2f_{RF}+\Delta f_{RF}\right)}^{2}}{2c}\right)\frac{L\lambda_{0}}{c}.$$

Formula (23) can be used to calculate, with high accuracy, the relative delay in the optical channel, especially when the optical chromatic dispersion coefficient of the path is characterized by a small value below 1 ps/nm*km.

**Figure 18:**
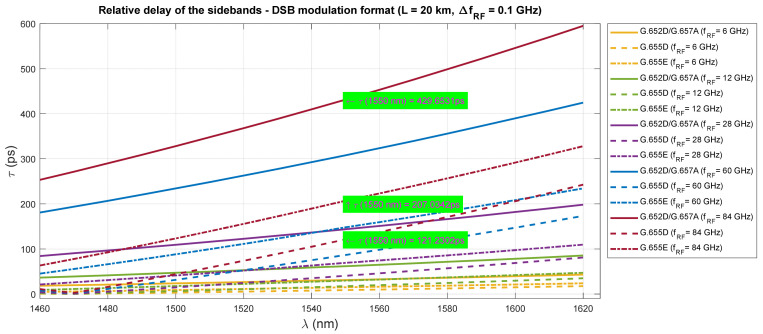
Calculation results of relative sideband delay in the optical channel (length of optical path/link is equal to 20 km and the radio channel frequency bandwidth for CP-OFDM modulation format is equal to 100 MHz).

The results of the relative delay of the sidebands are shown in Figure 18. The calculations were made for the fronthaul optical path with the maximum allowable length of 20 km. The optimal channel bandwidth was taken into account, i.e., 100 MHz, which is available in all bands from the FR1 and FR2 ranges, intended to be used in the 5G NR interface. The calculation results show that the relative delay is several orders less than the allowable relative multi-path delay over the radio link. The allowed relative delay value in the CP-OFDM modulated signal is governed by the duration of the cyclic prefix. In this case $T_{CP_{\min}}\gg \;\;\tau_{\max}$, so it can be concluded that the OFDM symbol used in the RFoF link is safe (Table 8). Figure 18 shows the calculation results for the optical carrier wavelength of 1550 nm and the radio carrier frequency of 84 GHz (this frequency is currently outside the range of FR2 but will likely be used in B5G/6G solutions). However, by testing the link parameters in the radio domain and by extended setting of the channel state information (CSI), this quantity should be taken into account. 

### 3.4. Optical Channel Selection Algorithm

**Figure 19:**
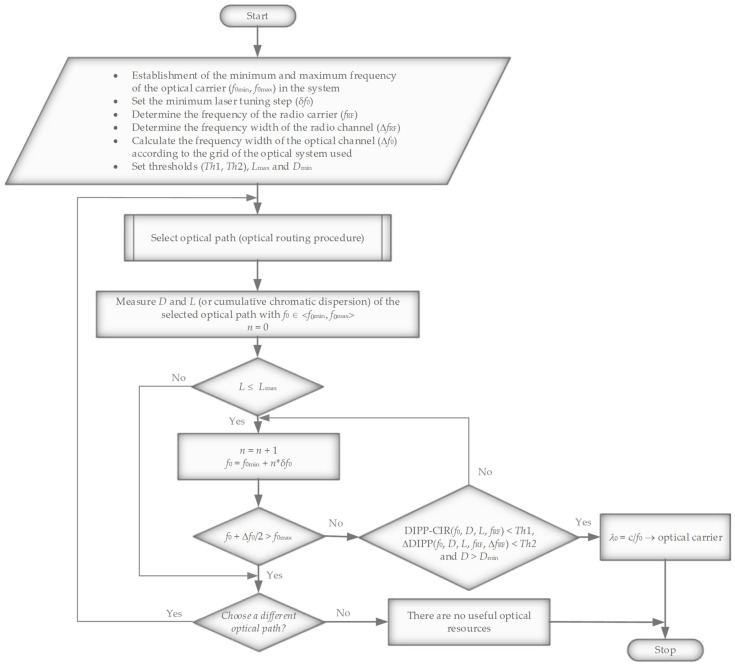
Optical channel selection algorithm based on DIPP-CIR calculations at two decision thresholds.

The proposed algorithm (Figure 19) assumes that the routing protocol working in the Xhaul will find available optical paths connecting the DU and RU. Thus, a set of possible connections on the optical layer should be available. It should be assumed that these paths are all-optical and transparent to signals modulated in the RFoF format. The first criterion excluding the path is its length *L*, which must be smaller than *L*max, because the permissible delay in the fronthaul link is of key importance. Further criteria are based on scanning free optical resources for the *D*max, *Th*1 and *Th*2 thresholds.

A first decision threshold, *Th*1, relates to the determination of an optical subband range that can be used for carrying the signal in the RFoF format. Two values were adopted here, i.e., 3 dB and 10 dB. In extreme cases, especially in the presence of a high radio frequency and a wideband frequency channel, an even stricter 1 dB criterion can be adopted. The second threshold, *Th*2, relates to the slope of the DIPP-CIR characteristic, which indicates how much they differ in the blanking level of the two outside subcarriers contained in the OFDM radio signal or physical resource block (PRB). The second threshold is especially important in cases where a wide frequency channel is used on a high carrier frequency with a high coefficient of chromatic dispersion present in the optical path.

The analysis of the phenomena occurring in the optical fronthaul path allowed for the construction of an algorithm (Figure 19) for the effective determination of the optimal optical channel for signal transmission in the DSB-RFoF format. Before the application created on the basis of the algorithm is launched, a lot of initial data must appear, which indicate not only the parameters of the radio signal, but also the properties of the optical path selected for transmission. The properties of the optical path include the chromatic dispersion coefficient *D* (or cumulative chromatic dispersion), path length *L* and its attenuation *A_pp_*. Due to the DIPP phenomenon occurring in a receiver operating in the DD mode, the most important parameter is the chromatic dispersion coefficient. This measurement can be performed with the use of the built-in OTDR system with the CD measurement function or with the use of an algorithm using optical AM modulation [51]. 

The introduction of the option to select an optical channel that meets the condition of minimum dispersion *D*_min_ greatly reduces the occurrence of cross-phase modulation (XPM) and four-wave mixing (FWM) phenomena. The value of the minimum chromatic dispersion coefficient is variable, as it depends on the type of optical signals multiplexed in the DWDM Xhaul link. In the case of a constant wavelength spacing between the channels in which radio signals with the same RF radio frequency are carried, the requirement for *D*_min_ is much higher. The paths/links of the DWDM Xhaul can transport the streams from the core network, backhaul, midhaul and fronthaul. Only on the fronthaul links can the RFoF signal be used. In other cases, it is a digital transmission in the OOK or coherent format. Interleaving in the wavelength domain of RFoF and OOK signals, with a flexible DWDM grid, further reduces the mutual influence of signals transported in adjacent optical channels. Please note that in the case of OOK modulation, the signal is concentrated close to the optical carrier, while in the RFoF signal, the sidebands containing the information are located at the periphery of the channel.

The capabilities of the transmitting laser indicate a jump in the tuning of the optical carrier and the maximum range of wavelength change. The main limitation here is the operating range of the optical system, where $f_{0}\in \langle f_{0\min},f_{0\max}\rangle$, including the ROADM nodal devices and optical fibers belonging to the selected optical path. During the calculations, it was assumed that in the DWDM system we do not exceed the narrowest single-modality range of optical fibers with non-zero chromatic dispersion shifted. The speed of tuning or switching the laser is decisive in terms of the final speed of selecting and setting up an optical channel. If no suitable optical channel is detected (Figure 12, Table 5), the process of selecting a new optical path must take place. This is related to the need to run a routing procedure, which was also included in the algorithm (Figure 19).

An EON or flexible DWDM network, e.g., an algorithm including routing, modulation level, and spectrum allocation (RMLSA) techniques [52,53,54], can be equipped with an additional computational-decision-making component based on the algorithm shown in Figure 19. This operation not only ensures precise selection of the optical path parameters in the case of a signal in the RFoF format, but can significantly improve the classical transmission in the on–off keying (OOK) format because fast streams also experience the DIPP phenomenon at the location of the photodetector, which lowers the sensitivity of the direct receiver.

## 4. Simulation Results

The above calculations can be confirmed by hardware experiments or computer simulation. In practice, the quality of the CP-OFDM radio signal transmitted over the optical path depends on many other factors that were not taken into account in the theoretical calculations. These include, among others, laser, modulator and photodetector parameters. Additionally, there is phase noise resulting from the influence of chromatic dispersion on the current phase state of individual OFDM subcarriers.

**Figure 20:**
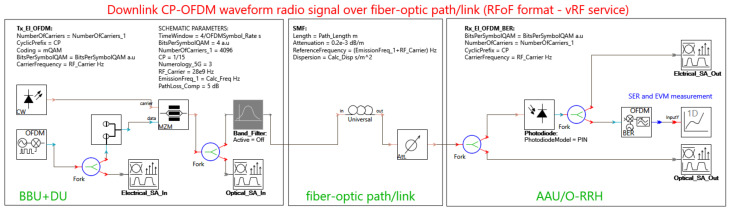
Simulation diagram prepared on the VPIphotonics Design Suite 11.1 platform, presenting three fronthaul parts, configured for downlink transmission: BBU + DU cloud side, fiber-optic path/link and AAU/O-RRH as an antenna side.

In order to confirm the correctness of the theoretical selection of optical subbands suitable for carrying the RFoF signal in the DSB format, the VPIphotonics Design Suite 11.1 simulation platform was used. This allowed for point-modeling of the quality of the CP-OFDM signal, carried by a specific radio and optical carrier, then demodulated in the direct detection format on the optical side, and in the coherent format on the radio side. The simulation scheme is shown in Figure 20.

The CP-OFDM signal was generated with the OFDM transmitter module. The signal structure was adopted according to the 3GPP-5G parameters given in Table 8, except for the number of subcarriers. A maximum number of 4096 (“NumberOfCarriers” parameter in Figure 20) was adopted to simplify the configuration options and to standardize the signal format for all radio frequency ranges. With this assumption, more difficult detection conditions arose due to the broadband signals. In practice, the simulator allows for switching off the subcarriers, but the variability of the planned guard intervals for individual radio bands would violate the constancy of the reference parameters.

The simulator performed calculations in the time of four CP-OFDM symbols (“TimeWindow” parameter—Figure 20). The value of this parameter was established by a compromise between the accuracy of the SER obtained and the time of the calculation. It should be noted that a fairly low level of the total loss of the 20 km path (0.2 dB/km) was established on the level of 9 dB with the attenuation components (“PathLoss_Comp” = 5 dB), which resulted in minimizing the impact of photodetector noise on the symbol condition. Thanks to this assumption, it was possible to demonstrate the characteristics of the SER dependent mainly on the DIPP phenomenon. It can be concluded here that any appearance of an additional attenuation component, e.g., a ROADM node in the passive optical path, will worsen these conditions. Additionally, it should be noted that the OFDM symbol is very energetic because its duration is due to the very low symbol rate (Table 8) occurring in the interfaces of cellular systems based on the OFDM modulation technique. The filling of the constellation is possible due to the large number of OFDM subcarriers, each of which is modulated separately, using modulation from the n-QAM family.

The CP-OFDM signal is upconverted to the RF carrier. Two carrier frequencies were selected for the simulation, i.e., 28 GHz and 60 GHz. To generate the optical carrier, a DFB laser emitting light with a power of 10 dBm and a spectral line of 10 kHz was used. During the simulation, the laser used was tuned in 100 GHz increments. The optical zero reference channel is located in the middle of the C band, and its central frequency is equal to 193.1 THz. The optical modulation process is carried out with the use of an external MZI-based modulator, the operating parameters of which were selected so that the RF radio signal is introduced onto the optical carrier in a linear manner. Light intensity modulation in MZI is performed correctly when the system operates in quadrature bias point (QP) configuration. This means that the optical MZM operates at a fixed operating point in the middle of the transmission characteristic. The modulated signal goes to a Gaussian bandpass filter, which is only turned on when the signal needs to be created in SSB format, for comparison purposes. The signal in the DSB format goes to the SMF, the length of which was set as the maximum, i.e., 20 km. In the fronthaul network, the optical path also cannot exceed this value, as reserved in the 3GPP recommendation.

Two types of optical fibers were selected for the simulation, i.e., G.652D/G.657A and G.655D. The calculation script developed on the basis of (1) and (2) determines the chromatic dispersion coefficient according to the current optical channel. The attenuation coefficient of the optical fiber is 0.2 dB/km. Additionally, an optical attenuator is used, which is a substitute for the optical loss components of the optical path according to Table 2. The default value of the attenuator is set to 5 dB. The optical signal is received by a PIN-type photo-receiver operating in the direct detection mode. This is where optical demodulation takes place. The RF radio signal goes to a coherent receiver, where it is demodulated to the baseband. This process takes place in an OFDM demodulator circuit, which is equipped with a software DSP. The options of automatic synchronization with the radio carrier and automatic normalization of the n-QAM constellation were enabled in the DSP. In practice, a separate synchronization line is needed, which provides an unmodulated radio carrier. The DSP is equipped with components enabling the determination of the SER and EVM. The SER reading, estimated by the Monte Carlo (MC) method, was used to evaluate the quality of the transmitted CP-OFDM signal.

**Figure 21:**
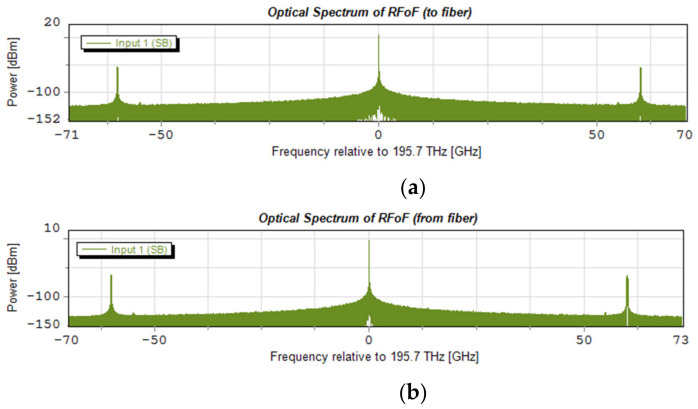
Example optical power spectrum of RFoF signal (*f*_RF_ = 60 GHz, G.655D fiber, optical channel no. 26 (195.7 THz), 4096 subcarriers, *µ* = 2): (**a**) inserted into the optical single-mode fiber/path, (**b**) at the photodetector input.

In order to be able to view the spectral characteristics, four spectrum analyzers were connected: two for monitoring the input and output optical signals and two for monitoring the RF electrical signals. Exemplary spectrograms of the optical side are shown in Figure 21. The spectrograms indicate the DSB format of the modulated optical signal, where the center frequency of the radio channel is 60 GHz. The optical carrier is 195.7 THz; it is the center of the optical channel number 26, which is located in the center of the optical subband to be simulated.

**Figure 22:**
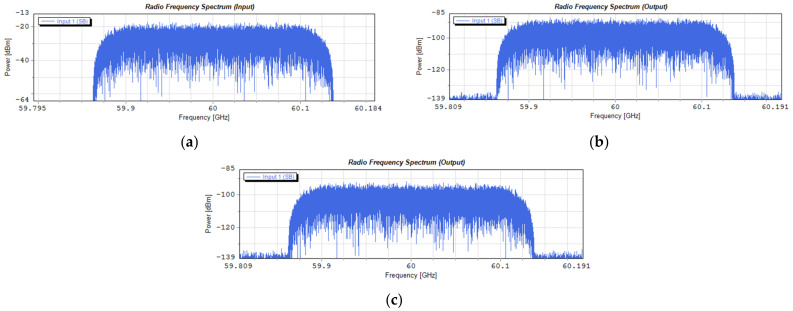
Example radio power spectrum of RF signal (*f*_RF_ = 60 GHz, 4096 subcarriers, *µ* = 2, 1.835 Gbps, Δ*f*_RF_ = 247.7 MHz): (**a**) input signal; (**b**) output signal transported over G.655D fiber in optical channel no. 26 (195.7 THz); (**c**) output signal transported over G.655D fiber in optical channel no. 14 (194.5 THz).

Figure 22 shows the spectrograms of radio signals that take place around a radio carrier of 60 GHz. The signal in this case was formed according to a numerological parameter of 2. Using 4096 subcarriers, a signal with a frequency width of approximately 247.7 MHz was created. This signal transports a 1.835 Gbps stream. Figure 22a shows the spectrum of the signal input to the optical modulator, and Figure 22b,c shows the spectra of the signal output from the photo-detector. Figure 22b shows the signal transported on the optical channel located in the center of the determined 10 dB subband according to *Th*1, and Figure 22c shows the spectrum of the signal transported on the optical channel located on the border of the determined 10 dB subband.

**Figure 23:**
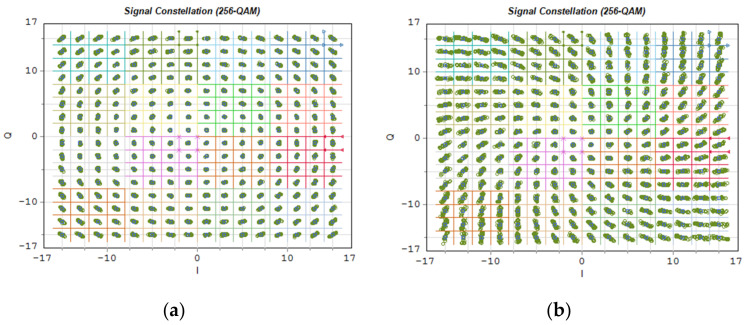
Example constellations of 256-QAM signal (*f*_RF_ = 60 GHz, G.655D fiber, *µ* = 2): (**a**) demodulated output signal transported over G.655D fiber in optical channel no. 26 (195.7 THz); (**b**) demodulated output signal transported over G.655D fiber in optical channel no. 14 (194.5 THz).

Figure 23 shows exemplary OFDM subcarrier constellations modulated in 256-QAM format. As in the case of Figure 22, Figure 23a shows the constellation of the radio signal carried on the optical channel no. 26, and Figure 23b, the constellation of the signal carried on the 10 dB outermost channel no. 14.

**Figure 24:**
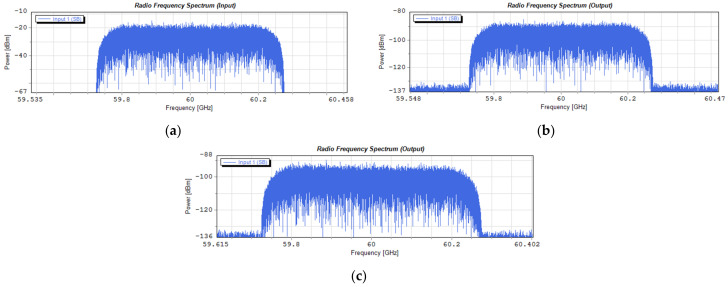
Example radio power spectrum of RF signal (*f*_RF_ = 60 GHz, 4096 subcarriers, *µ* = 3, *R_b_* = 2.752 Gbps, Δ*f*_RF_ = 491.5 MHz): (**a**) input signal; (**b**) output signal transported over G.655D fiber in optical channel no. 26 (195.7 THz); (**c**) output signal transported over G.655D fiber in optical channel no. 14 (194.5 THz).

Figure 24 shows the spectrograms of the radio signals introduced into the G.655D fiber and led out from the fiber. In this case, the radio signal was created on the basis of a numerological parameter with a value of 3, which resulted in the channel width increasing twice as compared to the signal presented in Figure 22.

**Figure 25:**
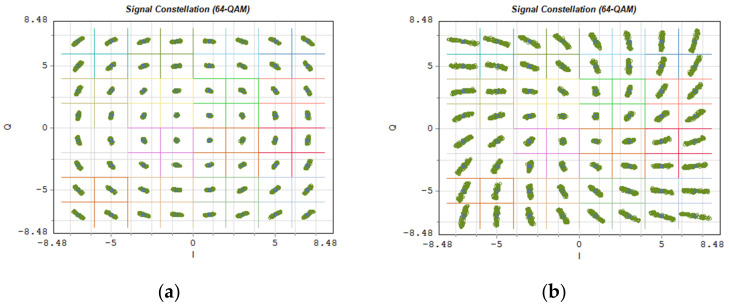
Example constellations of 64-QAM signal (*f*_RF_ = 60 GHz, G.655D fiber, *µ* = 3): (**a**) demodulated output signal transported over G.655D fiber in optical channel no. 26 (195.7 THz); (**b**) demodulated output signal transported over G.655D fiber in optical channel no. 14 (194.5 THz).

Figure 25 shows the constellations of a 64-QAM modulated signal on each OFDM subcarrier in the baseband. In this case, the signal comes from an OFDM demodulator to which 4096 subcarriers were provided on a frequency channel with a width of 491.5 MHz. Such a channel width indicates that a numerological parameter with a value of 3 was used. The propagation conditions in a single-mode fiber, in this case, are much more difficult. This manifests itself in the rapidly changing position of the constellation points in the interval, in the case of the optical channel located on the edge of the 10 dB optical band (Figure 25b).

**Figure 26:**
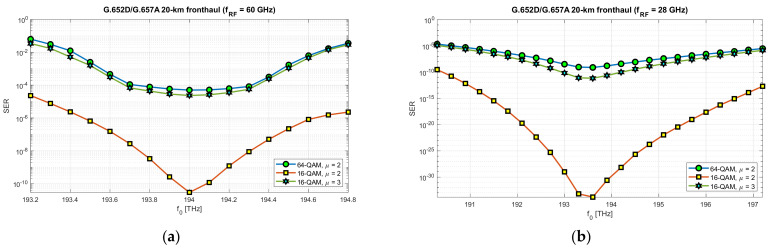
Simulation results presenting SER as a quality parameter of the selected CP-OFDM signals transported over the G.652D/G.657A single-mode fiber fronthaul (*L* = 20 km): (**a**) for radio carrier *f_RF_* = 60 GHz and the calculated optical 10 dB subband: 193.1630–194.7505 THz; (**b**) for radio carrier *f_RF_* = 28 GHz and the calculated optical 10 dB subband: 190.0130–197.2505 THz.

**Figure 27:**
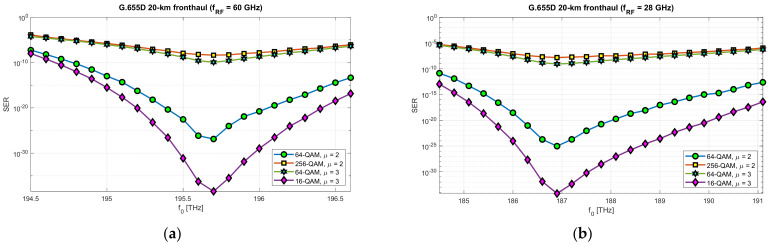
Simulation results presenting SER as a quality parameter of the selected CP-OFDM signals transported over the G.655D non-zero dispersion-shifted single-mode fiber fronthaul (*L* = 20 km): (**a**) for radio carrier *f_RF_* = 60 GHz and the calculated optical 10 dB subband: 194.5130–196.7255 THz; (**b**) for radio carrier *f_RF_* = 28 GHz and the calculated optical 10 dB subband: 184.4880–191.1068 THz.

In order to fully verify the correctness of the selection in the computational part of 10 dB optical subbands, suitable for transmitting radio signals in the RFoF format in the DSB version, simulations were carried out with the selected, previously mentioned, optical fibers and system parameters. The simulation results are presented in Figure 26 and Figure 27.

In the case of the optical path based on the G.652D/G.657A fiber, the radio signal was used in three modulation configurations with 16-QAM and 64-QAM modulation orders and a numerological parameter with a value of 2 and 3. Figure 27 shows the results of the simulation of radio signal transmission over an optical path based on the G.655D standard fiber. In this case, the tests were performed for 16-QAM, 64-QAM and 256-QAM modulation orders, with numerological parameters of 2 and 3. Not all combinations were tested, but the results obtained are sufficient to confirm the correct selection of optical subbands during the theoretical calculations.

## 5. Discussion

The Analog Option 10 interface, proposed to be introduced in the mobile fronthaul, working in the RFoF format (Figure 1), is a big challenge, but it can allow for a significant increase in the efficiency of using the network’s optical resources. This issue was widely analyzed and discussed in [21]. The idea of selecting an appropriate optical channel in order to transport the signal in analog form comes from the fact that the optical transmitter and receiver usually work in a DSB format. This means the work simplicity of both the modulator and the optical demodulator. The only component increasing the cost of fabrication of the transceiver is the option to tune the laser to the carrier wavelength selected using the proposed algorithm (Figure 19). To the best of the author’s knowledge, this type of approach in relation to the RFoF solutions has not been presented in the literature on the subject.

An external modulator is used in the simulation; however, in the case of a radio carrier with a lower frequency, the direct modulation technique can be used. This choice depends entirely on the laser frequency response. This results in an additional reduction in the costs of fabrication of the optical transmitter device. It is known that in the current 5G and future B5G/6G radio systems, MIMO techniques are and will be used, which means there is a need to provide more streams to the RRH antenna mast in order to properly radiate the beam (ABF and HBF techniques) and to use the spatial multiplexing technique. If we assume that the RF signal intended for a single antenna module is built in the cloud, then sending more such signals over the network will require a greater broadband. Fiber-optic networks make this possible because they use the xWDM techniques more and more often. The aforementioned optical Xhaul is also based on this.

The results of the calculations performed, presented in Section 3, show that this method may not be useful in all cases. Therefore, it is important to have a larger number of optical paths managed by network routing protocols (Figure 19). The optical sidebands interference phenomenon at the location of the photodetector is very strongly dependent not only on the chromatic dispersion of the optical path, but also on its length and the radio carrier frequency. Due to such a complex relationship, there are more dimensions that can be used to accurately select the optical resources. In Figure 6, Figure 7 and Figure 8, we can see how the fading effect of the sidebands signal increases with respect to the optical carrier as the carrier radio frequency increases. The periodicity of the DIPP interaction is also dependent on the optical fiber type used in the optical path. It should be assumed that the path may consist of various optical fibers (Table 3, Figure 5). Therefore, it is important to perform a test for its length and its averaged chromatic dispersion coefficient in accordance with the algorithm. Exemplary calculations were made for three types of optical fibers with the assumption that the fronthaul path reaches the maximum allowable length of 20 km and is made of one type of optical fiber. The results of the calculations shown in Figure 9, Figure 10 and Figure 11 are much more representative and illustrative. In addition to the three types of optical fibers, four radio carrier frequencies were used for the calculations. Not all the selected radio carrier frequencies are in the range FR1 or FR2. It is expected that the ranges indicated by 3GPP will be extended in the future. It is all about adding bands that are currently free from concession. In Figure 6a, Figure 7a and Figure 8a, we can see that the impact of DIPP on the 6 GHz frequency, after passing over the 20 km optical path, is negligibly small. On this basis, it was decided that the FR1 range would not be taken into account during further calculations, as very good conditions for receiving the DSB signal exist here. Carrier frequencies of 12, 28, 60 and 84 GHz were used for further calculations. Figure 9a, Figure 10a and Figure 11a show that only in the case of a long optical path based on the G.652D/G657A fiber may the transmission quality be lowered. Here, the choice of a longer wavelength of the optical carrier allows us to go in the range between 3 dB and 10 dB. For 28 GHz and 60 GHz radio frequencies, there is moderate decay periodicity, allowing us to find the right resources. However, we can find cases where we find ourselves on the border of the 10 dB range. Such a situation can, for example, be observed in the case of the G.652D path with a length of 5 km and the radio frequency of 28 GHz. In this case, all the optical carriers lying in the system band are in the range below the 10 dB threshold (Figure 9b). Such cases should be caught by an algorithm that requests an optical path change. Another problem is the frequent periodicity of fading. This is especially characteristic of the 84 GHz frequency that is transmitted over the G.652D/G.657A fiber. The range of white clearances is small, indicating a low number of optical channels that ensures high quality DSB signal transmission.

In the next stage of calculations, the process of determining the optical subbands for selected radio frequency carriers and the fronthaul path with a maximum length of 20 km was performed (Figure 12, Figure 13 and Figure 14). These types of charts allow us to see when there are no matching optical resources. Such a situation occurred, for example, in the case of the G.652D/G657A fiber and the 12 GHz frequency in the 3 dB access range (Figure 12b). On the basis of the performed calculations, the ranges of subbands for individual types of optical fibers and selected radio frequencies were generated. The results are listed in Table 5, Table 6 and Table 7. The values were recalculated and given in frequency to comply with the provisions used in the recommendations for the optical channel grid of the flexible DWDM system [37]. The analysis of the results presented in Table 5, Table 6 and Table 7 shows that in the case of optical fibers with a lower chromatic dispersion coefficient, the ranges are wider, which allows for the selection of an optical channel with a flat transmission characteristic of broadband signals. This is confirmed by the calculations, the results of which are presented in Figure 15, Figure 16 and Figure 17. Earlier calculations were performed for an unmodulated carrier. In the next stage of calculations, it was assumed that the radio signal is composed of a larger number of subcarriers, which is characteristic of the CP-OFDM format. The dispersiveness of the fiber causes different subcarriers to experience different relative delays. In the case of a large number of OFDM subcarriers in the radio signal, the channel bandwidth may reach 400 MHz. This value can be quickly achieved at higher values of the numerological parameter (Table 8). As can be expected, the greatest differential delay occurs in the case of optical fibers with a high chromatic dispersion coefficient, i.e., G.652D/G.657A (Figure 15). Here, we can observe ΔDIPP of a few dB. It is an important parameter, as it influences the correct functioning of the radio receiver in the scope of normalization of the constellation of the modulated signal in the n-QAM format in the area of the radio resource unit. A high value of this parameter results in the exclusion of a specific modulation order in the case of the need to transmit a radio signal in a specific optical channel based on a dispersive optical fiber.

In addition, calculations for the so-called multipath were made. The phenomenon of differential propagation of the optical sidebands of the DSB signal causes two replicas of the OFDM symbol to reach the receiver. The calculations are only a formality because the relative delay is several orders lower than the duration of the guard interval (Table 8). The results of the calculations are shown in Figure 18. However, when determining the parameters of the radio link between the DU and UE, this value should be taken into account as an addition to the channel state information (CSI) in a radio link.

In order to verify the possibility of transmitting the modulated RF signal over the optical path and the correct selection of optical subbands, a simulation was performed. Two types of single-mode optical fibers (G.652D/G.657A and G.655D) were used in the simulation and two radio frequency carriers were selected, i.e., 28 and 60 GHz. The simulation results showed that the optical subbands were calculated correctly (Figure 26 and Figure 27). Optical transmission of the DSB signal in the RFoF technique is burdened with many distortions, which was partially shown in the computational part of this study. The first limitation is the significant loss in the places where the signal is converted from electrical-to-optical (E/O) and optical-to-electrical (O/E). The results of the passive path loss can be seen in the pictured sample spectrograms (Figure 22 and Figure 24). It is a key component determining the overall level of the signal reaching the radio demodulator. The noise floor of the radio receiver and the photo-detector constitute the basic signal-to-noise ratio, which determines the quality of the received useful signal. It should be noted that there is no optical amplifier on the optical side, which is typical for short-range links. It should also be taken into account that, at the location of the RF signal receiver, before the signal is delivered to the antenna module, a radio amplifier should be used. However, the signal-to-noise ratio is crucial here. The simulation did not use linear and redundant information coding. The Monte Carlo (MC) method was used to assess the SER parameter. However, the results obtained are relative, as the SER value is strongly dependent on the parameters of optoelectronic systems and the cumulative loss of optical components in the optical path. Such components may include, for example, OADMs/ROADMs and splitters (Table 2). The simulation assumed that the total additional attenuation of optical components is 5 dB. An increase in this value proportionally increases the SER value over the entire range, as it lowers the signal-to-noise ratio, which is not due to the DIPP effect. This assumption is due to the fact that the classic optical components used in the optical path are not dispersive, with the exception of dedicated chromatic dispersion compensators. 

The constellations of the signals modulated in the 256-QAM and 64-QAM formats are shown in Figure 23 and Figure 25, respectively. Figure 23a and Figure 25a show constellations of the signal transported over the optical fiber in the most optimal optical channel in the designated optical subband. These constellations show the presence of phase noise that results from phase shifts due to optical sideband interference. This phenomenon intensifies with an increase in the chromatic dispersion coefficient and an increase in the radio carrier frequency. In this case, the increase in the radio frequency causes the frequency sidebands to recede, which in turn causes rapid changes in the resultant phase. The depicted constellations also show that, with low loss of the optical path (together with the optical components), the effect of the thermal noise of the photodetector is minimal. Figure 23b and Figure 25b show the constellations of the modulated signal carried in the optical channel at the edge of the 10 dB subband. In this case, the differences in the levels of adjacent subcarriers start to differ substantially. We observe this in the form of increasing amplitude noise in the presence of constant phase noise. External symbols (of greater amplitude) are most exposed to the increasing qualitative SER parameter, which is easy to observe. Figure 26 and Figure 27 show the simulation results as a compendium. In the case of the G.652D/G.657A fiber, the simulation of the modulated signal in the 256-QAM format was abandoned, as phase noise causes too much reduction in the transmission quality. It is possible but with a significant reduction in the radio carrier or the bandwidth of the frequency channel. The simulations were performed for frequencies within the FR2 range and above this range, where it was not planned to use a numerological parameter with a value below 2. The simulation results also show that the transmission quality is significantly influenced by the bandwidth of the frequency channel, and more specifically, the numerological parameter of the CP-OFDM signal. For *µ* = 3, the subcarrier spacing increases to 120 kHz, which greatly increases the distance differences between the levels of the same numbered interfering subcarriers in the optical sidebands. Additionally, with the increasing value of the parameter *µ*, the length of the OFDM symbol is shortened, which reduces its energy. The total consequences can be seen in Figure 26, where we compare the SER value for 16-QAM modulation, with the *µ* parameter being 2 and 3. Increasing the *µ* parameter by one degree results in a SER similar to 64-QAM for *µ* = 2. This applies to both the 60 GHz and 28 GHz radio carriers.

The same phenomenon is observed in the case of the G.655D fiber, although in this case, the results are much better, due to the lower chromatic dispersion coefficient. Here, 256-QAM modulation for *µ* = 2 was introduced, due to its much better dispersion parameters. Confusingly, Figure 27a,b shows swapped values. In the case of the 28 GHz radio carrier, the SER values are slightly higher than in the case of the 60 GHz carrier. However, one should look at the subband in which the simulations were performed. The optical subband for 60 GHz is characterized by higher frequencies, i.e., shorter wavelengths, so here, there is a lower chromatic dispersion coefficient. In the case of 28 GHz, the optical frequencies are much lower, so the optical wave is longer at a higher value of the chromatic dispersion coefficient. 

It is undisputed that the introduction of redundant coding will significantly increase the quality of the transmitted digital information. However, the strict quality characteristics presented in Figure 26 and Figure 27 clearly show which parameters of the radio signal must be taken into account when we are selecting an optical channel. For lower radio frequencies, the available optical subbands are much wider. This results in a lot of freedom in the selection of optical channels, and in such a channel, the amplitude characteristics are much flatter.

The discussion presented above concerned the research performed on the basis of the well-known standards of single-mode optical fibers, which are designed and manufactured with the use of silica. These types of optical fibers have become very entrenched in telecommunications fiber-optic networks, so breaking this barrier will certainly not be easy. A very significant disadvantage of standard single-mode optical fibers is the small diameter of the core, in which optical signals appearing in the adjacent optical channels very quickly increase the power density. This fact causes the appearance of the above-mentioned non-linear effects, which can significantly deteriorate the transmission. The chromatic dispersion occurring in, and in the vicinity of, the core, which is the essential limiter of DSB-RFoF signal transport, is important from the point of view of limiting the Kerr effect, which results in XPM and FWM distortions. In conclusion, these two phenomena should be balanced so that communication is effective. If we want to stick to this technology, we need to popularize multi-core fibers much faster [55]. Fibers based on a greater number of cores are more difficult to couple with the transmitter and receiver systems, but they allow for a significant spatial increase in cable capacity. In the B5G/6G wireless access systems, the massive-MIMO technique will be the norm. Connecting a large number of antenna modules, located on the RU/RRH mast, to the BBU, also via the DU, requires the use of a large number of optical fibers heavily loaded with high optical power density. This is the specificity of the C-RAN architecture described in the introduction. Therefore, it is indisputable that the popularization of multi-core optical fibers in distribution and access networks will significantly simplify the management of often terabit fronthaul traffic.

## 6. Conclusions and Future Work

The proposed new analog interface, with the acronym Option 10, could apply to next-generation wireless systems. The condition for introducing this solution is the development of optical Xhaul networks working in the flexible grid DWDM format. Optical nodal devices must be transparent due to the possibility of carrying RFoF signals. The A-RoF technique is more spectrally effective than D-RoF solutions and allows the use of simple AAU/RRH devices. However, the transmission of analog signals over optical fibers causes many problems. The attenuation of silica fibers is very low, but they have dispersion that must be taken into account. The paper presents a method of using the optical path to transport the DSB-RFoF radio signal in which no chromatic dispersion compensation is used. However, it is indisputable that static or dynamic compensation of chromatic dispersion is an unrivaled but costly solution. Chromatic dispersion pre-compensation can improve the total dispersion balance, which will allow the transport of RFoF signals with a higher radio carrier frequency, also exceeding 84 GHz. This may be particularly important when the fiber-optic access path lengths are long in relation to the links between the Xhaul nodes. The access fibers are usually of the G.652D or G.657A standard, which in the DWDM system operating band, introduces a high chromatic dispersion coefficient (examples of variants are given in Table 3 and Figure 5). SSB transmission can also be used, which, however, is associated with the need to use tunable filters or dedicated single-sideband modulation systems. The proposed method of selection of an optical channel based on the flexible DWDM grid for transporting RF radio signals between the DU and RU/RRH, over a dispersive optical fiber, may be a good complement to the existing techniques. This can be especially useful when we need to mass-produce transceivers for optical fronthaul communications.

The calculations and simulations were carried out on the assumption that only one modulated RF signal is transported in the selected optical channel. In practice, in an optical Xhaul link working in the flexible DWDM format, there may be more optical channels located close to each other. There may be a high power density in the core of the optical fiber. In such a case, one should take into account the possibility of the above-mentioned non-linear distortions causing the interaction of signals located in adjacent optical channels. This is one of the issues that will be discussed in further research. In the case of multi-channel, also we need to pay attention to the guard spacing between channels. This will entail the need to disable OFDM subcarriers as required by the system requirements of the 3GPP recommendation. In this paper, this element was omitted in order to simplify the structure of the CP-OFDM signal.

## Figures and Tables

**Table 1:** Empirical and usually measured single-mode optical fiber attenuation coefficients [44].

Fiber	1310 nm	1380 nm	1550 nm	1625 nm
G.652D/G.657A—std	0.33 dB/km	0.31 dB/km	0.20 dB/km	0.25 dB/km
G.652D/G.657A—max ^1^	0.40 dB/km	0.4 dB/km	0.3 dB/km	0.4 dB/km
G.655D/E—std	-	-	0.22 dB/km	0.27 dB/km
G.655D/E—max ^2^	-	-	0.35 dB/km	0.4 dB/km

^1^ According to recommendations [41,42]. ^2^ According to recommendation [43].

**Table 2:** Example loss/attenuation of passive components in the optical fronthaul path.

Path Component	Fiber (*α*)	Splice (*a_s_*)	Connector APC/UPC (*a_c_*)	ROADM Add-Drop (*a_ad_*)	ROADM Passthrough (*a_pt_*)	Splitter (*a_s_*)
Loss (dB)	-	0.05–0.1	0.1–0.3	1.5–3	1–4	3.5–20
Attenuation (dB/km)	0.2–0.4 ^1^	-	-	-	-	-

^1^ Changing with wavelength according to Figure 2.

**Table 3:** Examples of the optical paths with different standards of the single-mode optical fibers used in individual internodal links.

Variant No.	Link A		Link B		Link C	
	Fiber	Length (km)	Fiber	Length (km)	Fiber	Length (km)
1	G.652D	5	G.655D	12	G.652D	3
2	G.652D	3	G.655E	10	G.657A	2
3	G.657A	7	G.655D	8	G.652D	3
4	G.657A	10	G.655E	3	G.657A	7
5	G.652D	1	G.655D	17	G.657A	1

**Table 4:** Optical channel bandwidths for selected RF carriers in the vRF service (RFoF interface). Δ*λ*_0_ is given for an optical carrier with a wavelength equal to 1550 nm.

*f_RF_* (GHz)	6	12	28	60	84
Δ*f*_0_ (GHz)	12.5	25	100	200	200
Δ*λ*_0_ (nm)	~0.1	~0.2	~0.8	~1.6	~1.6

**Table 5:** Calculated availability of optical subbands for two thresholds in a 20-km fronthaul path designed on the basis of G.652D or G.657A single-mode fiber.

*f_RF_* (GHz)	Optical Subband (THz)	
	3-dB	10-dB
6	205.3380–184.4880	205.3380–184.4880
12	-	205.3380–198.8568
28	195.6068–191.4130	205.3380–203.4067; 197.2505–190.0130 ^1^;
		185.6130–184.4880
60	203.7692–202.5880; 200.3817–199.3067;	204.2067–202.1693; 200.7817–198.9193;
	197.2817–196.2880; 194.4130–193.4942;	197.6442–195.9317; 194.7505–193.1630 ^1^;
	191.7505–190.8942; 189.2692–188.4692;	192.0692–190.5880; 189.5630–188.1817;
	186.9442–186.1942; 184.7630–184.4880	187.2255–185.9255; 185.0255–184.4880
84	204.8630–204.2442; 203.0505–202.4567;	205.0942–204.0192; 203.2630–202.2442;
	201.3130–200.7505; 199.6567–199.1192;	201.5255–200.5443; 199.8568–198.9255;
	198.0755–197.5567; 196.5505–196.0567;	198.2630–197.3692; 196.7318–195.8755;
	195.0880–194.6130; 193.6817–193.2255;	195.2630–194.4380; 193.8505–193.0567;
	192.3255–191.8817; 191.0192–190.5942;	192.4942–191.7255; 191.1755–190.4380;
	189.7567–189.3442; 188.5380–188.1380;	189.9130–189.1942; 188.6880–187.9942;
	187.3568–186.9692; 186.2130–185.8380;	187.5005–186.8317; 186.3505–185.7067;
	185.1068–184.7442	185.2380–184.6130

^1^ selected optical subband for simulation.

**Table 6:** Calculated availability of optical subbands for two thresholds in a 20 km fronthaul path designed on the basis of G.655D non-zero dispersion shifted single-mode fiber.

*f_RF_* (GHz)	Optical Subband (THz)	
	3-dB	10-dB
6	205.3380–184.4880	205.3380–184.4880
12	205.3380–187.8630	205.3380–184.4880
28	205.3380–200.5755; 189.2442–184.5630	205.3380–198.1880; 191.1068–184.4880 ^1^
60	205.0192–203.3630; 200.3505–198.9067;	205.3380–202.7880; 200.8880–198.3942;
	196.2442–194.9630; 192.5630–191.3817;	196.7255–194.5130 ^1^; 193.0067–190.9630;
	189.1817–188.1130; 186.1130–185.1317	189.5817–187.7318; 186.4755–184.7880
84	204.6068–203.7630; 202.1693–201.3880;	204.9130–203.4630; 202.4630–201.1068;
	199.9005–199.1693; 197.7755–197.0880;	200.1693–198.9067; 198.0255–196.8380;
	195.7692–195.1192; 193.8817–193.2630;	196.0130–194.8880; 194.1068–193.0380;
	192.0567–191.4567; 190.3130–189.7505;	192.2755–191.2442; 190.5255–189.5443;
	188.6567–188.1192; 187.0817–186.5630;	188.8568–187.9255; 187.2692–186.3817;
	185.5692–185.0817	185.7505–184.9005

^1^ selected optical subband for simulation.

**Table 7:** Calculated availability of optical subbands for two thresholds in a 20 km fronthaul path designed on the basis of G.655E non-zero dispersion shifted single-mode fiber.

*f_RF_* (GHz)	Optical Subband (THz)	
	3-dB	10-dB
6	205.3380–184.4880	205.3380–184.4880
12	205.3380–194.0192	205.3380–184.4880
28	195.5130–190.1505	197.5317–188.3255
60	203.2130–201.7692; 199.1130–197.8317;	203.7505–201.2630; 199.5942–197.3755;
	195.4505–194.2880; 191.9880–190.8255;	195.8755–193.8817; 192.4193–190.4130;
	188.6567–187.5942; 185.6130–184.6380	189.0443–187.2192; 185.9692–184.4880
84	204.9317–204.1567; 202.6755–201.9505;	205.2192–203.8755; 202.9442–201.6880;
	200.5567–199.8755; 198.5630–197.9130;	200.8130–199.6255; 198.8005–197.6817;
	196.6693–196.0567; 194.8755–194.2943;	196.8942–195.8380; 195.0942–194.0817;
	193.1442–192.5317; 191.3505–190.7630;	193.3755–192.3067; 191.5630–190.5505;
	189.6380–189.0817; 188.0067–187.4755;	189.8442–188.8817; 188.2005–187.2817;
	186.4505–185.9442; 184.9567–184.4880	186.6380–185.7567; 185.1380–184.4880

**Table 8:** Selected parameters of CP-OFDM modulation used in the 5G New Radio interface [49,50].

5G Numerology (*µ*)	BB-Bandwidth (Δ*f_RF_*)	Subcarrier Spacing	OFDM Subcarriers	Modulation Order (min–max)	Symbol-Rate	1-Layer Bit Rate (max)	FFT Symbol Duration	CP Duration (*T*_CP_)	FFT + CP Duration
-	(MHz)	(kHz)	-	-	(Bd)	(Mbps)	(µs)	(µs)	(µs)
0	5	15	300	2–256	14,000	33.60	66.67	4.69	~71.36
0	10	15	624	2–256	14,000	69.89	66.67	4.69	~71.36
0	20	15	1272	2–256	14,000	142.46	66.67	4.69	~71.36
0	50	15	3240	2–256	14,000	362.88	66.67	4.69	~71.36
1	80	30	2604	2–256	28,000	583.30	33.33	2.34	~35.67
1	100	30	3276	2–256	28,000	733.82	33.33	2.34	~35.67
2	200	60	3168	2–256	56,000	1419.26	16.67	1.17	~17.84
3	400	120	3168	2–256	112,000	2838.53	8.33	0.57	~8.90
4	400	240	1536	2–256	224,000	2752.51	4.17	0.29	~4.46

## Data Availability

All data are included within manuscript.

## References

[1] Camps-Mur D., Gutierrez J., Grass E., Tzanakaki A., Flegkas P., Choumas K., Giatsios D., Beldachi A.F., Diallo T., Zou J. (2019). 5G-XHaul: A novel wireless-optical SDN transport network to support joint 5G backhaul and fronthaul services. IEEE Commun. Mag..

[2] (2017). 5G-XHaul, D2.3. Architecture of Optical/Wireless Backhaul and Fronthaul and Evaluation. https://www.5g-xhaul-project.eu/download/.

[3] 5GPPP, Architecture Working Group (2020). View on 5G Architecture. https://5g-ppp.eu/wp-content/uploads/2020/02//.

[4] Cooper A.J. (1990). Fiber/radio for the provision of cordless/mobile telephony services in the access network. Electron. Lett..

[5] Lee C.H. (2013). Microwave Photonics.

[6] (1999). ITU-R, F.1332-l. Radio-Frequency Signal Transport through Optical Fibres. https://www.itu.int/rec/R-REC-F.1332/.

[7] (2015). ITU-T, Series G, Supplement 55. Study Group 15. Radio-over-Fibre (RoF) Technologies and Their Applications. https://www.itu.int/rec/T-REC-G.Sup55/.

[8] (2015). CPRI Industry Forum (Ericsson, Huawei, NEC, and Nokia). CPRI Specification 7.0. www.cpri.info.

[9] Pfeiffer T. (2015). Next generation mobile fronthaul and midhaul architectures. J. Opt. Commun. Netw..

[10] Zakrzewski Z. Fronthaul optical networks working with use of the hybrid analog and digital radio-over-fiber techniques. Proceedings of the International Society for Optical Engineering, 17th Conference on Optical Fibers and Their Applications.

[11] (2019). CPRI Industry Forum (Ericsson, Huawei, NEC, and Nokia). eCPRI Specification 2.0. www.cpri.info.

[12] (2019). IEEE 1914.1. Standard for Packet-Based Fronthaul Transport Networks. https://standards.ieee.org/standard/1914_1-2019.html.

[13] (2017). 3GPP TR 38.801 v14.0.0. 03.2017. Study on New Radio Access Technology: Radio Access Architecture and Interfaces. https://www.3gpp.org/ftp//Specs/archive/38_series/38.801/38801-e00.zip.

[14] (2019). IEEE, 1588-2019. IEEE Standard for a Precision Clock Synchronization Protocol for Networked Measurement and Control Systems. PNCS—Precise Networked Clock Synchronization Working Group. https://standards.ieee.org/standard/1588-2019.html.

[15] (2019). ITU-T, G.8261. Timing and Synchronization Aspects in Packet Networks. https://www.itu.int/rec/T-REC-G.8261.

[16] (2018). ITU-T, G.8262. Timing Characteristics of Synchronous Ethernet Equipment Slave Clock. https://www.itu.int/rec/T-REC-G.8262.

[17] (2018). IEEE, P802.1CM. IEEE Standard for Local and Metropolitan Area Networks. Time-Sensitive Networking for Fronthaul..

[18] Ng B.L. Fulfilling the promise of massive MIMO with 2D active antenna array. Proceedings of the IEEE Globecom Workshops.

[19] (2020). O-RAN Alliance. Use Cases and Overall Architecture Workgroup. O-RAN Architecture Description 3.0. Technical Specification ORAN-WG1. https://www.o-ran.org/specification-access.

[20] Zakrzewski Z. Optical RRH working in an all-optical fronthaul network. Proceedings of the 8th International Conference on Photonics, Devices and Systems, SPIE—The International Society for Optical Engineering.

[21] Zakrzewski Z. (2020). D-RoF and A-RoF interfaces in an all-optical fronthaul of 5G mobile systems. Appl. Sci..

[22] Meslener G.J. (1984). Chromatic dispersion induced distortion of modulated monochromatic light employing direct detection. IEEE J. Quantum Electron..

[23] Ih C.S., Gu W. (1990). Fiber induced distortions in a subcarrier multiplexed lightwave system. IEEE J. Select. Areas Commun..

[24] Schmuck H. (1995). Comparison of optical millimeter-wave system concepts with regard to chromatic dispersion. Electron. Lett..

[25] Elrefaie A.F., Lin C. Clipping Distortion and Chromatic Dispersion Limitations for 1550 nm Video Trunking Systems. Proceedings of the IEEE Symp. Computers and Communications.

[26] Gliese U., Nielsen S.N., Nielsen T.N. Limitations in distance and frequency due to chromatic dispersion in fiber-optic microwave and millimeter-wave links. Proceedings of the IEEE MTT-S Int. Microwave Symp. Dig..

[27] Park J., Elrefaie A.F., Lau K.Y. (1996). Fiber chromatic dispersion effects on multichannel digital millimeter-wave transmission. IEEE Photon. Technol. Lett..

[28] Fuster J.M., Marti J., Corral J.L., Polo V., Ramos F. (2000). Generalized study of dispersion-induced power penalty mitigation techniques in millimeter-wave fiber-optic links. J. Lightwave Technol..

[29] Marti J., Fuster J.M., Laming R.I. (1997). Experimental reduction of chromatic dispersion effects in lightwave microwave/millimetre-wave transmissions using tapered linearly chirped fibre gratings. Electron. Lett..

[30] Ilgaz M.A., Batagelj B. Using tunable dispersion-compensated modules to overcome the power penalty of a millimeter-wave opto-electronic oscillator signal that is distributed via a passive optical network for 5G networks. Proceedings of the 11th International Symposium on Communication Systems, Networks & Digital Signal Processing (CSNDSP).

[31] Ilgaz M.A., Baliž K.V., Batagelj B. (2020). A flexible approach to combating chromatic dispersion in a centralized 5G network. Opto-Electron. Rev..

[32] Smith G.H., Novak D., Ahmed Z. (1997). Technique for optical SSB generation to overcome fiber dispersion penalties in fiber-radio systems. Electron. Lett..

[33] Won P., Zhang W., Williams J. Self-phase modulation dependent dispersion mitigation in high power SSB and DSB + dispersion compensated modulated radio-over-fiber links. Proceedings of the MTT-S International Microwave Symposium Digest.

[34] Yaakob S., Mahmood R.M., Zan Z., Rashidi C.B.M., Mahmud A., Anas S.B.A. (2021). Modulation index and phase imbalance of dual-sideband optical carrier suppression (DSB-OCS) in optical millimeter-wave system. Photonics.

[35] Ishimura S., Kim B.G., Tanaka K., Nishimura K., Kim H., Chung Y.C., Suzuki M. (2018). Broadband IF-over-fiber transmission with parallel IM/PM transmitter overcoming dispersion-induced RF power fading for high-capacity mobile fronthaul links. IEEE Photonics J..

[36] Ishimura S., Bekkali A., Tanaka K., Nishimura K., Suzuki M. (2018). 1.032-Tb/s CPRI-equivalent rate IF-over-fiber transmission using a parallel IM/PM transmitter for high-capacity mobile fronthaul links. J. Lightwave Technol..

[37] (2020). ITU-T, G.694.1. Spectral Grids for WDM Applications: DWDM Frequency Grid. https://www.itu.int/rec/T-REC-G.694.1/.

[38] (2021). O-RAN Alliance. O-RAN Open X-haul Transport WG9. WDM-based Fronthaul Transport 1.0. Technical Specification ORAN-WG9. https://www.o-ran.org/specification-access.

[39] Zakrzewski Z. Transport of Rel-15/16 waveform radio signals over optical 5G fronthaul path. Proceedings of the International Society for Optical Engineering, 19th Conference on Optical Fibers and Their Applications.

[40] (2018). ITU-T, GSTR-TN5G. Transport Network Support for IMT2020/5G. https://www.itu.int/dms_pub/itu-t/opb/tut/.

[41] (2016). ITU-T, G.652. Characteristics of a Single-Mode Optical Fibre and Cable. https://www.itu.int/rec/T-REC-G.652/.

[42] (2016). ITU-T, G.657. Characteristics of a Bending-Loss Insensitive Single-Mode Optical Fibre and Cable. https://www.itu.int/rec/T-REC-G.657/.

[43] (2009). ITU-T, G.655. Characteristics of a Non-Zero Dispersion-Shifted Single-Mode Optical Fibre and Cable. https://www.itu.int/rec/T-REC-G.655/.

[44] (2020). ITU-T, G.650.1. Definitions and Test Methods for Linear, Deterministic Attributes of Single-Mode Fibre and Cable. https://www.itu.int/rec/T-REC-G.650.1/.

[45] Elrefaie A.F., Wagner R.E., Atlas D.A., Daut D.G. (1988). Chromatic dispersion limitations in coherent lightwave transmission systems. J. Lightwave Technol..

[46] Lim C., Nirmalathas A., Bakaul M., Lee K.L., Novak D., Waterhouse R. (2009). Mitigation strategy for transmission impairments in millimeter-wave radio-over-fiber networks. J. Opt. Netw..

[47] (2021). 3GPP TR 38.101-1 v17.2.0. 06.2021. User Equipment (UE) Radio Transmission and Reception. Part 1: Range 1 Standalone (Release 17). https://www.3gpp.org/ftp/Specs/archive/38_series/38.101-1/38101-1-h20.zip.

[48] (2021). 3GPP TR 38.101-2 v17.2.0. 06.2021. User Equipment (UE) Radio Transmission and Reception. Part 2: Range 2 Standalone (Release 17). https://www.3gpp.org/ftp/Specs/archive/38_series/38.101-2/38101-2-h20.zip.

[49] (2021). 3GPP TR 38.211 v16.6.0. 06.2021. NR. Physical Channels and Modulation (Release 16). https://www.3gpp.org/ftp/Specs/archive/38_series/38.211/38211-g60.zip.

[50] (2021). 3GPP TR 38.104 v17.2.0. 07.2021. NR. Base Station (BS) Radio Transmission and reception (Release 17). https://www.3gpp.org/ftp/Specs/archive/38_series/38.104/38104-h20.zip.

[51] Devaux F., Sorel Y., Kerdiles J.F. (1993). Simple measurement of fiber dispersion and of chirp parameter of intensity modulated light emitter. J. Lightwave Technol..

[52] Christodoulopoulos K., Tomkos I., Varvarigos E. (2011). Elastic bandwidth allocation in flexible OFDM-based optical networks. J. Lightwave Technol..

[53] Christodoulopoulos K., Tomkos I., Varvarigos E. Spectrally/bitrate flexible optical network planning. Proceedings of the 36th Eur. Conf. Exhibit. Opt. Commun..

[54] Tomkos I., Azodolmolky S., Solé-Pareta J., Careglio D., Palkopoulou E. (2014). A tutorial on the flexible optical networking paradigm: State of the art, trends, and research challenges. Proc. IEEE.

[55] Zakrzewski Z. (2013). Microwave-photonic networks based on single-mode multi-core optical fibers. Photonics Lett. Pol..

